# A P-Glycoprotein Is Linked to Resistance to the *Bacillus thuringiensis* Cry3Aa Toxin in a Leaf Beetle

**DOI:** 10.3390/toxins8120362

**Published:** 2016-12-05

**Authors:** Yannick Pauchet, Anne Bretschneider, Sylvie Augustin, David G. Heckel

**Affiliations:** 1 Department of Entomology, Max Planck Institute for Chemical Ecology, Hans-Knoell-Str. 8, Jena 07745, Germany; abretschneider@ice.mpg.de (A.B.); heckle@ice.mpg.de (D.G.H.); 2 Unité de Zoologie Forestière, Institut National de la Recherche Agronomique (INRA), 2163 Avenue de la Pomme de Pin, CS 40001 Ardon, Orléans 45075 CEDEX 2, France; sylvie.augustin@inra.fr

**Keywords:** ABC proteins, Bt Cry3Aa toxin, *Chrysomela tremula*, leaf beetle, Bt resistance

## Abstract

*Chrysomela tremula* is a polyvoltine oligophagous leaf beetle responsible for massive attacks on poplar trees. This beetle is an important model for understanding mechanisms of resistance to *Bacillus thuringiensis* (Bt) insecticidal toxins, because a resistant *C. tremula* strain has been found that can survive and reproduce on transgenic poplar trees expressing high levels of the Cry3Aa Bt toxin. Resistance to Cry3Aa in this strain is recessive and is controlled by a single autosomal locus. We used a larval midgut transcriptome for *C. tremula* to search for candidate resistance genes. We discovered a mutation in an ABC protein, member of the B subfamily homologous to P-glycoprotein, which is genetically linked to Cry3Aa resistance in *C. tremula*. Cultured insect cells heterologously expressing this ABC protein swell and lyse when incubated with Cry3Aa toxin. In light of previous findings in Lepidoptera implicating A subfamily ABC proteins as receptors for Cry2A toxins and C subfamily proteins as receptors for Cry1A and Cry1C toxins, this result suggests that ABC proteins may be targets of insecticidal three-domain Bt toxins in Coleoptera as well.

## 1. Introduction

Crystal (Cry) toxins produced during sporulation by the Gram-positive bacterium *Bacillus thuringiensis* (Bt) are highly potent against insects and for many years have been successfully used as biopesticides in agriculture. The main advantage of Cry toxins relies on their narrow spectrum compared to more traditional broad-spectrum chemical insecticides such as organochlorines, synthetic pyrethroids, and organophosphates. Indeed, different Cry toxins are highly specific to certain insect orders such as Lepidoptera, Diptera and Coleoptera [1]. The exponential increase in planting insect-resistant crop plants transformed to express Bt-derived insecticidal Cry proteins has enabled a substantial reduction in the use of chemical insecticides [2]. However, it has also increased the selection pressure for target insects to develop resistance to these Bt crops. For example, the western corn rootworm has recently developed resistance in the field to several transgenic maize lines expressing different Bt Cry toxins [3,4]. Therefore, efforts directed to understand the mode of action of Bt Cry toxins in insects and the associated resistance mechanisms are crucial to develop efficient crop pest management strategies.

The leaf beetle, *Chrysomela tremula* Fabricius (Coleoptera: Chrysomelidae) is an important model for understanding the mode of action of Bt toxins and Bt resistance in Coleoptera because a Cry3Aa-resistant *C. tremula* strain was selected on Bt-transformed poplar trees expressing the Cry3Aa toxin [5]. This strain was derived from an isofemale line established from field-caught insects that generated F2 offspring that survived on this Bt poplar clone [5]. This was unexpected because the original field-caught insects used to generate the Cry3Aa-resistant strain did not experience any human-induced selection pressure; indeed, these Bt poplars have not been disseminated in France and the Cry3Aa toxin has never been used in French pest management [5]. The resistance ratio of this isofemale line was estimated to be more than 6400 compared to a susceptible *C. tremula* strain (LC_50_ = 31.1 ng purified Cry3Aa/cm^2^ leaf surface), allowing Cry3Aa-resistant insects to complete their life cycle on Bt poplars [5]. Resistance to Cry3Aa in *C. tremula* is under control of a single, almost completely recessive, autosomal trait [6], suggesting that changes in a single receptor, or other gene product, may be involved in resistance.

Here we report on the identification of the gene responsible for Cry3Aa resistance in *C. tremula* combining a candidate gene approach, genetic linkage analyses and heterologous protein expression in insect cells. This gene encodes an ABC transporter in the B subfamily, homologous to P-glycoprotein, which we named CtABCB1. We demonstrate that the resistance to Cry3Aa in *C. tremula* is linked to the occurrence of a four-base-pair deletion in the open reading frame of CtABCB1 in resistant insects, and that insects homozygous for the presence of this deletion are resistant to Cry3Aa. We also provide evidence that CtABCB1 may act as a receptor to Cry3Aa in *C. tremula*. This work represents a crucial step in understanding the detailed mode of action of the Cry3Aa toxin in Coleoptera and is of considerable significance for the management of Bt resistance globally.

## 2. Results

### 2.1. A Four-Base-Pair Deletion in CtABCB1 Is Genetically Linked to Cry3Aa Resistance

We used a larval midgut transcriptome for *C. tremula* [7] to identify candidate genes for resistance to Cry3Aa. Based on the mode of action of Bt Cry toxins in Lepidoptera, we examined gene families encoding ABC proteins, cadherin-like proteins, aminopeptidases N (APNs) and alkaline phosphatases as potential candidates [8,9]. A previous report indicated that, in *C. tremula*, there was no difference in sequence and in expression of three APNs between insects of the susceptible and the resistant strains [10]. We turned to ABC proteins because of their association with resistance to Cry1A and Cry2A toxins in Lepidoptera [11,12,13,14]. We used a recent analysis of the tissue-specific expression of genes encoding ABC proteins in *C. populi*, a sister species of *C. tremula*, to identify ABC proteins expressed in the larval midgut. The CpABC12 gene of *C. populi* had the highest expression and encoded a full transporter of the B subfamily [15]. We obtained the full-length cDNA sequence of the *C. tremula* homolog which we named CtABCB1 (GenBank Accession GU462154), which shared more than 90% amino acid identity with CpABC12.

**Figure 1:**
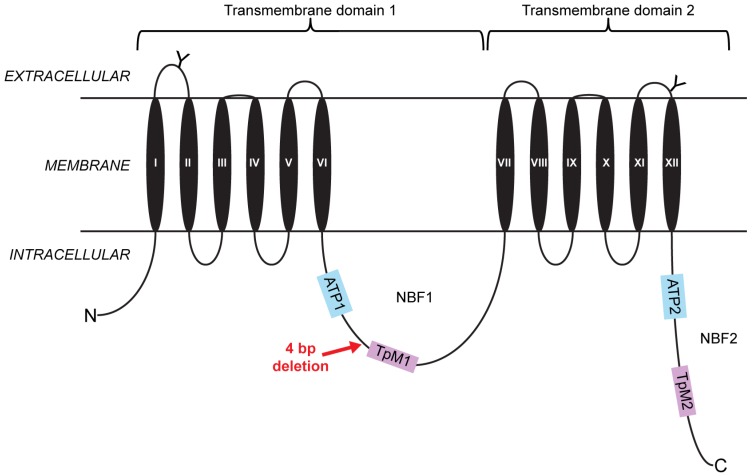
Diagram of the CtABCB1 protein structure and location of the mutation present in resistant *C. tremula* individuals. Predicted glycosylation sites on two of the extracellular loops are represented by “Y.” Two highly conserved ATP nucleotide binding folds (NBF1, NBF2) that include the transporter signature motifs 1 and 2 (TpM1, TpM2) are present in the intracellular environment. The structure of CtABCB1consists of two transmembrane domains (TMD 1, TMD 2), each of them made of six transmembrane helices (TM I-VI in TMD 1; TM VII-XII in TMD 2). The approximate position of the four-base-pair deletion discovered in resistant individuals is indicated by a red arrow.

The open reading frame (ORF) of CtABCB1 is 3780 bp long (Figure S1) and encodes a protein of 1259 amino acids possessing all general features of full-transporter ABC proteins (Figure 1 and Figure S2), such as two transmembrane domains each composed of six transmembrane helices, and two nucleotide binding folds (NBF1 and 2) each composed of an ATP binding domain (ATP) and a transporter motif (TpM1 and 2). We then PCR-amplified the ORF of CtABCB1 from larval midgut cDNAs prepared from Cry3Aa-resistant insects. These showed a four-base-pair deletion at position 1561 (GenBank Accession KX686490, Figure S1), introducing a frame shift with a premature stop codon leading to loss of the TpM1 transporter motif as well as the complete second transmembrane domain (Figure 1). A homolog of CtABCB1 in the western corn rootworm, *Diabrotica virgifera virgifera*, was shown to be genetically linked to resistance to the Bt Cry3Bb1 toxin; however, the resistance-conferring mutation was not reported [16] (Figure S4). The existence of cross-resistance between Cry3Bb1 and mCry3A (a modified version of Cry3Aa) in the western corn rootworm [17] suggested that CtABCB1 could be involved in resistance to Cry3Aa in *C. tremula*, and we investigated it further.

**Figure 2:**
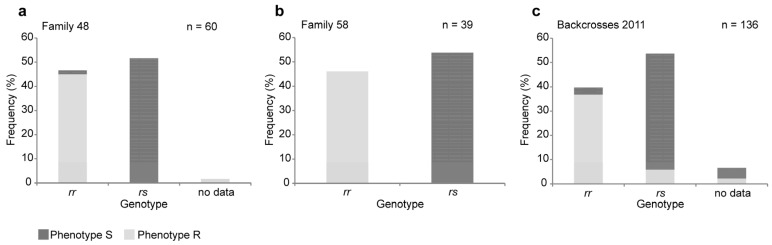
Genotyping of the mutation in CtABCB1 in backcrosses between susceptible and resistant individuals. Crosses (mating pairs) between individuals of the susceptible and the resistant strains were set up in 2015 (panels **a**,**b**) and in 2011 (panel **c**). The progeny of these crosses (F1) were backcrossed to individuals of the resistant strains also in mating pairs. (**a**) Phenotype and genotype for backcross family 48; (**b**) Phenotype and genotype for backcross family 58; (**c**) Phenotype and genotype for the backcrosses set up in 2011 which correspond to the offspring from seven backcross families having all the same pair of grandparents but different pairs of parents. The offspring of these backcrosses were selected for four days on leaves of Bt poplars. During this time, individuals found dead were considered susceptible to Cry3Aa (phenotype S) and the ones that survived and actively fed were considered resistant to Cry3Aa (phenotype R). Genotyping of each individual was performed by amplifying by PCR the region where the deletion was discovered followed by Sanger sequencing. Individuals with genotype “*rr*” are homozygous for the presence of the four-base-pair deletion on CtABCB1, whereas individuals with genotype “*rs*” are heterozygous for the presence of this mutation. “No data” indicates that the genotyping did not work, neither at the PCR level nor at the sequencing level.

We set up two sets of single-pair crosses between the susceptible and resistant strains, the first in Orléans in early 2011 and the second in Jena in late 2015. The F1 progeny were backcrossed to the resistant strain in single-pair crosses. Backcross progeny were selected for four days on leaves of Bt poplar. Individuals found dead were considered susceptible to Cry3Aa (phenotype S) and the ones that survived and actively fed were considered resistant to Cry3Aa (phenotype R). DNA was isolated from all R as well as S progeny, and examined for segregation of the four-base-pair deletion in CtABCB1. Progeny were either *rr* (with two copies of the four-base-pair deletion) or *rs* (heterozygous, with one copy of the four-base-pair deletion and one copy of the wild-type allele). Overall, 44% of the progeny were *rr* and 56% were *rs* (Figure 2, Dataset S1), and this ratio was not significantly different from the 50:50 ratio expected according to Mendelian inheritance (G = 2.78, df = 1, *p* > 0.1) and not significantly different across the three families (G_H_ = 0.45, df = 2, *p* > 0.7). The CtABCB1 genotype was strongly associated with survivorship on Bt poplar overall (Figure 2, G = 194.98, df = 2, *p* < 0.0001), with nonsignificant differences among families (G_H_ = 0.003, df = 2, *p* > 0.9). For crosses performed in 2015, 99% of the progeny were either *rr* and R (surviving on Bt poplar), or *rs* and S (killed by Bt poplar)—a nearly perfect correlation (Figure 2, Dataset S1). For crosses performed in 2011, the correlation was somewhat lower with 91% of progeny being either *rr* and R, or *rs* and S. Altogether, these results provide strong evidence that the four-base-pair deletion in CtABCB1 is genetically linked to Cry3Aa resistance in *C. tremula*, although minor genetic or environmental factors may also affect survivorship on Bt poplar.

### 2.2. Lepidopteran Insect Cells Expressing CtABCB1 Are Susceptible to Cry3Aa

*Sf*9 cells derived from *Spodoptera frugiperda* have previously been used to study the role of the ABCC2 proteins from *Bombyx mori* and *Heliothis virescens* as receptors for Cry1A toxins [18,19]. *Sf*9 cells do not express ABCC2 itself, the cadherin-like protein, aminopeptidases N or alkaline phosphatases [18]; moreover, expressing a coleopteran-derived protein in this lepidopteran cell system should reduce the risk of interference from other putative Cry toxin receptors even more.

**Figure 3:**
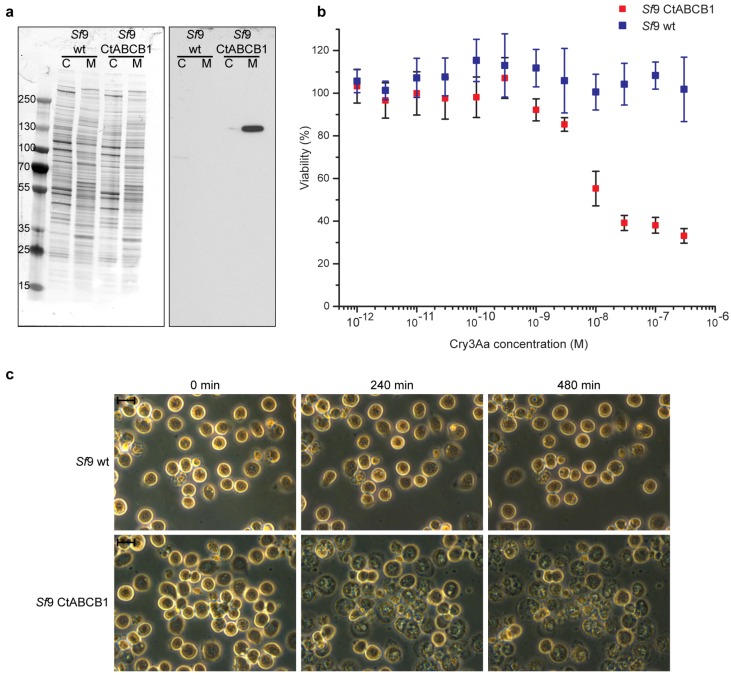
Heterologous expression of CtABCB1 in insect *Sf*9 cells. (**a**) Western blot with a V5 epitope-specific antiserum of both cytosoluble fraction (C) and crude membrane fraction (M) prepared from untransfected and transfected *Sf*9 cells; (**b**) Effect of the Cry3Aa toxin on cell viability (±SD). Trypsin-activated Cry3Aa was used in concentrations ranging from 10^−12^ M to 3.10^−7^ M and cells were treated for 24 h. Blue squares: untransfected *Sf*9 cells. Red squares: CtABCB1-expressing *Sf*9 cells. The data are based on a MTT assay (*N* = 6). Values over 100% are due to increase in cell number due to cell division over time in the untransfected *Sf*9 cells; (**c**) Morphological changes of *Sf*9 cells treated with 30 nM trypsin-activated Cry3Aa. Cells were observed for eight hours and pictures were taken every two hours. Scale bars: 10 µm.

We succeeded in isolating and expanding a clonal *Sf*9 cell line expressing CtABCB1 which originated from a single transformed cell. To confirm that CtABCB1 was properly expressed and translocated to the plasma membrane, we isolated both a crude membrane extract and a cytosolic fraction from these cells, and checked the expression of CtABCB1 by Western blot using an antibody directed against a V5 epitope cloned in frame at the carboxyl terminus of CtABCB1 (Figure 3A). A signal corresponding to CtABCB1 of approx. 130 kDa was only detected in the crude membrane fraction of the transformed clonal cell line and not untransfected *Sf*9 cells, close to the estimated size of this ABC protein (138.9 kDa).

Treatment with trypsin-activated Cry3Aa revealed a concentration-dependent decrease of viability of cells expressing CtABCB1 after 24 h of incubation (Figure 3B). In contrast, no decrease of viability could be detected for untransfected *Sf*9 cells (Figure 3B). However, Cry3Aa did not kill 100% of the CtABCB1-expressing cells, as viability could only be reduced to approximately 30%. A similar effect was obtained on *Sf*9 cells co-expressing the *H. virescens* cadherin-like protein and ABCC2 after treatment with either Cry1Aa or Cry1Ab or Cry1Ac, whereby the viability could only be reduced to 20% to 30% according to the toxin used [18].

Microscopic observation of CtABCB1-expressing cells treated with 30 nM of trypsin-activated Cry3Aa toxin showed dramatic morphological changes such as swelling, granule formation and lysis, but not for untransfected *Sf*9 cells (Figure 3C). These changes occurred relatively slowly, only after several hours. This is in contrast to previous studies on ABCC2 in Lepidoptera [18,19], with morphological changes evident after less than an hour of toxin treatment on ABCC2-expressing cells. We see three possible explanations to these observations. First, the expression of CtABCB1 that was achieved in our stable clonal cell line may be lower than the expression of lepidopteran ABCC2 in *Sf*9 cells. Second, other proteins in *C. tremula* besides ABCB1 may enhance the toxicity of Cry3Aa, but these were not expressed in the *Sf*9 cells. For example, cadherin-like proteins have been reported as potential functional receptors of Cry3Aa and Cry3Bb toxins in the beetles *Tenebrio molitor* and *Alphitobius diaperinus* [20,21]. Third, the activation of proCry3Aa to Cry3Aa using trypsin may not be optimal compared to the use of other proteases, beetle gut juice or beetle brush border membrane vesicle preparations, possibly reducing its toxicity [22,23]. Nonetheless, our results indicate that CtABCB1 is capable of mediating pore formation and cell swelling caused by Cry3Aa, major features of the mode of action of Bt toxins.

## 3. Discussion

We have described a major mechanism of resistance to Bt toxins in Coleoptera. In contrast to Lepidoptera, reports on Bt resistance in Coleoptera are relatively rare. A strain of the Colorado potato beetle *Leptinotarsa decemlineata* was selected with Cry3A, attaining 59-fold resistance [24], and higher survivorship of second instar larvae and adults on transgenic Cry3A-expressing potato plants [25]. A Cry3Aa-selected strain of the cottonwood leaf beetle *Chrysomela scripta* was >9000-fold resistant to Cry3Aa, 400-fold cross-resistant to Cry1Ba, but susceptible to Cyt1Aa [26]. As previously mentioned, an F2 screen of *C. tremula* from Vatan, France produced three resistant lines and an estimate of 0.0036 for the frequency of the resistant allele [5]. A later study using one of these resistant lines (#60) in an F1 screen of samples from Bar-de-Luc, 400 km away, yielded an even higher estimate of 0.011 [27]. Although these studies illustrated the potential for resistance to Bt poplar, there had not been any prior selection pressure in the field by these transgenic plants, although the amount of selection by Bt in the natural environment is unknown. The first report of field-evolved resistance in a coleopteran pest was in the western corn rootworm. *D. virgifera virgifera,* which caused feeding damage on Cry3Bb1-expressing maize fields in Iowa in 2009 [3]. Some of these fields had been planted with Cry3Bb1 or Cry34/35Ab1-expressing maize since 2004. The latest reports indicate extensive resistance and cross-resistance patterns among Cry3Bb1, mCry3A and eCry3.1Ab, but not to Cry34/35Ab1-expressing maize so far [17,28]. Although developing later than in Lepidoptera, Bt resistance in Coleoptera threatens to be just as significant a problem for agriculture [29].

The few studies on the mode of action of pore-forming Bt toxins and resistance mechanisms in Coleoptera are in general agreement with the more extensive studies in Lepidoptera. Pore formation by the toxin is enhanced upon activation by native brush border membrane vesicles of *Leptinotarsa* [30], likely due to a membrane-associated ADAM metalloprotease [31]. Other changes in protease composition are correlated with Cry3Aa resistance in the same species [32]. A cadherin protein similar to the Cry1A-binding cadherin of Lepidoptera has been identified in *Diabrotica* [33]. Similar to previous results with Lepidoptera [34], fragments of this cadherin synergize Cry3Aa and Cry3Bb activity against *Diabrotica* and *Leptinotarsa* [35] and the lesser mealworm, *Alphitobius diaperinus* [36], and a similar result was found for the cadherin from the mealworm, *Tenebrio molitor* [20]. The demonstration of genetic linkage between an ABC protein and Cry3Bb1 resistance in *D. virgifera* [16] was the first confirmation from Coleoptera of similar results in Lepidoptera [12,37]. In addition to a linkage analysis, our results add the molecular identity of the mutation in *C. tremula*, and a demonstration of the role of the CtABCB1 protein in cell killing by the Cry3Aa toxin. These studies suggest important similarities in the mode of action of Bt toxin among different species of Coleoptera.

The ABC proteins identified in *Diabrotica* and *C. tremula* are homologs of mammalian P-glycoprotein (MDR1 or ABCB1) [38], which has been intensively studied in toxicology and cancer biology because of its ability to confer resistance to chemotherapy by exporting a huge variety of compounds out of the cell [39]. These are full-transporters belonging to the B subfamily of ABC proteins and are expressed in the plasma membrane at the cell surface. Other members of the B subfamily are half-transporters internally localized in the endoplasmic reticulum, mitochondria or lysosome. The model coleopteran, *Tribolium castaneum*, has only two of these full-length B subfamily transporters in its genome, named TcABCB-3A and TcABCB-3B [40]. These occur on different chromosomes, and ABC-B proteins from other Coleoptera are similar to one or the other (Figures S4 and S5). The ABC-B protein linked to Cry3Bb1 resistance in *Diabrotica* as well as CtABCB1 is more similar to TcABCB-3B (Figure S4). The function of these ABC transporters in beetles is unknown, although by analogy to P-glycoprotein function in mammals, they are likely to export xenobiotics as well as endogenous compounds from cells. Interest in the role of P-glycoproteins in protecting organisms against chemical pesticides is increasing [41]. In a comprehensive RNA inhibition screen of all of the ABC proteins in *Tribolium*, no obvious phenotypic effects were seen by RNAi of TcABCB-3A or TcABCB-3B, in contrast to severe developmental defects and lethality seen by RNAi of the half-transporter TcABCB-5A [40]. Therefore, similar to the situation in Lepidoptera, certain full-length ABC proteins may be useful but not essential for survival of coleopteran pests in the field.

Results on fitness costs of Bt resistance in Coleoptera are mixed. Studies with Cry3Bb1-resistant laboratory strains of *D. virgifera* feeding on non-transgenic maize showed either a fitness benefit [42] or costs and benefits in different fitness components [43]. In experiments with the Bar-le-Duc-resistant strain of *C. tremula* studied by Wenes et al. [27], the frequency of the recessive resistant allele declined from 0.5 to 0.179 over five generations of rearing on non-Bt poplar, indicating a fitness cost of resistance. This strain must also have been carrying mutations in the same CtABCB1 gene that we studied, because it was isolated using the F1 screen with the same resistant isofemale line (#60) from Vatan. Thus, incapacitating mutations in coleopteran ABCB genes may have a fitness cost that could be exploited to combat Bt resistance.

In Lepidoptera, an ABC protein facilitates the entry of the pore into the plasma membrane [12,44], after binding to a cadherin which promotes pre-pore formation [45]. When the ABCC2 protein is heterologously expressed in otherwise toxin-insensitive cell lines, toxin-mediated pore formation, swelling and lysis occurs [18,19,46]. Expression of other Bt-toxin binding proteins such as aminopeptidase [47,48,49] or cadherin [18,19] has a much weaker effect. Recently, several mutations in ABCA2, a member of the A subfamily of ABC proteins, were found to confer high resistance against Cry2Ab1 in two Lepidoptera, *Helicoverpa armigera* and *H. punctigera* [13]. Our study adds a third subfamily of ABC proteins and a different toxin, active against Coleoptera but not Lepidoptera, to this interaction. These similarities suggest a common mechanism of pore insertion in lepidopteran and coleopteran-active toxins, involving ABC proteins.

We propose that this common mechanism could support the rational design of alternatives to combat the growing problem of Bt resistance by coleopteran pests. Maize expressing beetle-active Cry toxins is widely planted in the USA, and the inadequacy of current preventive resistance management strategies has been pointed out [50,51]. Bt-expressing poplars have been commercialized in China, and are expected to be widely adopted there [52]. In both systems, an unexpectedly high frequency of pre-existing resistance alleles would make resistance prevention very difficult. Proactive strategies that target the common resistance mechanism by increasing its fitness cost would become more attractive. One such strategy has been suggested by Xiao et al. [46], who found that Bt-resistance-causing mutations in the ABCC2 protein of *H. armigera* made the insects more susceptible to certain chemical insecticides. If mutations in ABC proteins are a common Bt-resistance mechanism in Coleoptera, a similar strategy may be useful in prolonging the utility of beetle-active toxins for control of this important group of pests.

## 4. Materials and Methods

### 4.1. Insect Rearing and Genetic Crosses

Cry3Aa-susceptible and Cry3Aa-resistant *Chrysomela tremula* larvae and beetles were obtained from field collections from Vatan, France [5]. (Earlier publications on these strains used *tremulae* as the species name instead of *tremula*). The susceptible strain originated from the offspring of an isofemale line that lacked alleles conferring resistance to the Cry3Aa toxin [5]. The resistant strain was established from an isofemale line (#60) selected on the foliage of hybrid poplars (*Populus tremula* × *Populus tremuloides*) and then genetically engineered to express a synthetic Cry3Aa gene derived from the native *Bacillus thuringiensis var. tenebrionis* [53]. This strain was fixed for an autosomal recessive allele conferring resistance to the Cry3Aa toxin [6]. Beetles were maintained in standard rearing conditions, in a growth chamber at 20 °C with a photoperiod of 16:8 (L:D). Larvae and adults were reared on fresh leaves detached from greenhouse-grown poplar hybrid clones that did not express Cry3Aa. Three-day-old third-instar larvae were used for dissection and further RNA isolation.

Grandparents—for example, a male from the susceptible strain and a female from the resistant strain—were mated, and their offspring (F1) reared to adulthood on detached leaves from control poplar hybrid clones. An F1 female was mated to a second male from the resistant strain (parents) and the resulting backcross offspring were reared for seven days on foliage from control poplar hybrid clones. Early third-instar larvae from the backcross offspring were then put individually on leaf discs from Cry3Aa-expressing hybrid poplars in 12-well plates for four consecutive days. Survival on Cry3Aa-expressing poplar was recorded every day. As soon as a larva was found dead, it was immediately collected and frozen at −80 °C. At the end of the four-day period, surviving larvae were also collected and frozen at −80 °C and considered as being resistant to Cry3Aa. Grandparents and parents of these crosses were also collected and frozen at −80 °C for further analyses. Note that these crosses were performed in both directions for grandparents and parents.

### 4.2. Genotyping of the Crosses

PCR primers were designed to flank the region of CtABCB1 where the four-base-pair deletion found in resistant individuals was located (Table S1). These primers were designed to possess either a M13_F or M13_R “tail” at their 5′-end for further Sanger sequencing. Genomic DNA was isolated from each individual from the backcross offspring as well as from the grandparents and parents using a “salting out” method as described by Martinez-Torres et al. [54]. Standard PCR reactions were performed in a thermocycler Mastercycler ep gradient S (Eppendorf AG, Hamburg, Germany) using the following parameters: initial denaturation at 95 °C for 1 min; 35 cycles of 95 °C for 15 s, 55 °C for 30 s and 72 °C for 30 s; final extension step was at 72 °C for 5 min. PCR products were inspected on 1.5% agarose gels before being cleaned up using the DNA Clean and Concentrator-5 kit (Zymo Research Europe, Freiburg, Germany). Sanger sequencing was carried out on an ABI 3730xl DNA Analyzer (Applied Biosystems, Foster City, CA, USA). The resulting sequencing chromatographs were inspected individually and genotypes were assessed as described in Figure S3. Results of the phenotyping and genotyping of the backcrosses as well as data analysis are summarized in Dataset S1. Data analysis employed G-statistics as described by Sokal and Rohlf [55]). Trace files are available in Supplementary Dataset S2 (family 48), Supplementary Dataset S3 (family 58) and Supplementary Dataset S4 (backcrosses 2011) which can be downloaded at [56].

### 4.3. Expression of CtABCB1 in Sf*9* Cells

*Spodoptera frugiperda*-derived *Sf*9 cells were cultured in Sf-900II serum-free medium (Gibco, Thermo Fisher Scientific, Waltham, MA, USA) supplemented with 50 µg/mL Gentamicin (Invitrogen, Thermo Fisher Scientific) at 27 °C.

Total RNA extraction from larval midgut of *C. tremula* was performed using the innuPrep RNA Mini kit (Analytik, Jena, Germany). RNA was treated with Turbo DNAse (Ambion, Thermo Fisher Scientific) and cleaned up with the RNeasy MinElute cleanup kit (Quiagen, Hilden, Germany). For first-strand cDNA synthesis 900 ng RNA were used and processed using the Verso cDNA kit (Thermo Fisher). The full-length CtABCB1 (NCBI: GU462154) cDNA sequence was amplified by PCR (primers: see Table S1) before being ligated in pIB/V5-His TOPO TA and used for stable transfection of *Sf*9 cells.

*Sf*9 cells were plated in 60 mm tissue culture dishes (Falcon, Corning, NY, USA) at approx. 70% confluency and transfected using FUGENE (Promega, Madison, WI, USA). Selection of cells was started 48 h post-transfection. Cloning cylinders (Sigma Aldrich, Munich, Germany) as well as limiting dilution series were applied to obtain cell clones expressing CtABCB1. Conditioned medium (the supernatant of exponentially growing three- to four-day-old *Sf*9 cells) supplemented with 10% (*v*/*v*) of heat-inactivated fetal bovine serum (FBS; Gibco) was used to support cell colony growth. For selection of clonal cell lines, culture medium was supplemented with 50 µg/mL Blasticidin (Invitrogen).

### 4.4. Western Blotting

Cells were plated in T75 flasks. At 100% confluency, cells were washed and harvested in phosphate buffered saline (PBS). The total cellular membrane proteins were extracted (Plasma Membrane Protein Extraction Kit, abcam, Cambridge, UK) and the concentration was determined by Bradford assay. Three micrograms of each sample were used. Samples were heated at 55 °C for 5 min and separated by SDS-PAGE (Criterion Precast gels, BioRad, Munich, Germany) and transferred to Immuno-Blot PVDF membrane (BioRad). Membranes were blocked in 1× Tris buffered saline (TBS, BioRad) supplemented with 0.2% Tween 20 (Sigma Aldrich) and 5% *w*/*v* milk powder (Roth, Karlsruhe, Germany) for 1 h at room temperature. Blots were then incubated with an anti-V5-HRP antibody overnight at 4 °C (Invitrogen). Bound antibodies were detected using an in-house detection solution (100 mM Tris-HCl pH 8.5, 90 mM coumaric acid, 250 mM luminol, 0.04% H_2_O_2_).

### 4.5. Toxin Preparation, Viability Assays and Morphological Changes

*Bacillus thuringiensis var. tenebrionis* carrying the gene-encoding Cry3Aa was obtained from the *Bacillus* Genetic Stock Center (Ohio State University). Cry3Aa protoxin was prepared according to Carroll et al. [22], and was activated with trypsin at a trypsin/protoxin ratio of 1/100 (*w*/*w*) at 37 °C for 2 h before further purification by anion exchange chromatography using a 1 mL RESOURCE Q column (GE Healthcare, Freiburg, Germany).

*Sf*9 cells were plated in 96-well cell culture plates (flat bottom, Greiner bio-one cellstar) at approx. 60% confluency. Cry3Aa (10^−12^ M–3.10^−7^ M) solubilized in 50 mM Na_2_CO_3_ pH 9.5 was added directly to the culture medium and cells were incubated for 24 h at 27 °C. The reaction volume was 100 µL. As control (0 nM Cry3Aa), we added a maximum of 3% of the buffer in the culture medium corresponding to the highest amount of buffer used for the dilution series of the toxin. The culture medium was removed and replaced with culture medium containing 0.5 mg/mL thiazolyl blue tetrazolium blue bromide (Sigma Aldrich) to perform an MTT assay. After 2 h of incubation at 27 °C, the medium was removed and replaced by 50 µL dimethyl sulfoxide (DMSO, Sigma Aldrich). Subsequently, the 96-well plates were briefly vortexed to dissolve the formazan crystals, and absorbance was measured at 540 nm (Infinite m200, Tecan, Maennedorf, Switzerland). All values were calculated in relation to untreated cells (defined as 100%). Six replicates were performed per treatment on each cell line (*Sf*9 untransfected and CtABCB1-expressing *Sf*9 cells). For the observation of morphological changes, cells were plated in 60 mm petri dishes. Cells were incubated with 30 nM of Cry3Aa and were observed for 8 h on a Zeiss Axiovert200 microscope. A picture was taken every 120 min with an AxioCam MrC5 camera and further processed with the program AxioVision AC (Release 4.3 (11-2004)).

## Supplementary Materials

The following are available online at www.mdpi.com/2072-6651/8/12/362/s1, Figure S1: Comparison between CtABCB1 cDNA sequences derived from either the susceptible or the resistant populations, Figure S2: Predicted protein sequence of CtABCB1, Figure S3: Determination of the genotype for the backcrosses between susceptible and resistant *C. tremula*, Figure S4: Neighbor-joining tree of full-transporter ABCB protein sequences from Coleoptera, Figure S5: CLUSTAL Alignment of ABCB protein sequences from Coleoptera, Table S1: Primers used in this study and their function, Dataset S1: Details of the phenotyping and genotyping of the backcrosses, Dataset S2: Trace files corresponding to the genotyping of family 48, Dataset S3: Trace files corresponding to the genotyping of family 58, Dataset S4: Trace files corresponding to the genotyping of “backcrosses 2011.”
