# Supplementary material for: A P-Glycoprotein Is Linked to Resistance to the Bacillus thuringiensis Cry3Aa Toxin in a Leaf Beetle
Source: Toxins (Basel). 2016 Dec 5;8(12):362. doi: 10.3390/toxins8120362 (PMC5198556; doi:10.3390/toxins8120362)
Supplement: Supplementary file 1 [file toxins-08-00362-s001.zip › toxins-153443-suppl-done/toxins-153443-supplementary done.pdf]

A P-Glycoprotein Is Linked to Resistance to the *Bacillus thuringiensis* Cry3Aa Toxin in a Leaf Beetle

Yannick Pauchet, Anne Bretschneider, Sylvie Augustin and David G. Heckel

Table S1. Primers used in this study and their function.

Primer Name	Sequence (5′–3′)	Function
CTR_ABCB1_del_F	TGTAAAACGACGGCCAGTAAAGACCTCGACCTCACCTG	Genotyping
CTR_ABCB1_del_R	CAGGAAACAGCTATGACCGAAGTGGCTTCGTCCAAGAG	Genotyping
M13_F	TGTAAAACGACGGCCAGT	Genotyping/ sequencing
M13_R	CAGGAAACAGCTATGACC	Genotyping/ sequencing
CTR_ABCB1_F	***ACC***ATG***G***GGAAACGTGAAAAATATTC	Cloning pIB/ V5-His TOPO/TA
CTR_ABCB1_R	AGTTGTCTGTCTTTGCAACTTAT	Cloning pIB/ V5-His TOPO/TA

The M13 "tails" of the primers used for the genotyping are underlined. The Kozak sequence introduced on the forward primer used to amplify *CtABCB1* for cloning in the expression vector is indicated in bold/italic.

```
                           10        20        30        40        50        60        70
CTR_ABC-B1_S.seq    ATGGGGAAACGTGAAAAATATTCATTGGATAAAAAAAATAAGAGCCCATTAGACGTCGAATTCACTAAAAATGAA
CTR_ABC-B1_R.seq    ATGGGGAAACGTGAAAAATATTCATTGGATAAAAAAAATAAGAGCCCATTAGACGTCGAATTCACTAAAAATGAA

                      80        90       100       110       120       130       140
CTR_ABC-B1_S.seq    GAAAAAGAAGGAGATAAGACCAAACAAGTATCTTTTTTTCAAATGTTCAGGTATGCCACAGGATTCGACAAGCTC
CTR_ABC-B1_R.seq    GAAAAAGAAGGAGATAAGACCAAACAAGTATCTTTTTTTCAAATGTTCAGGTATGCCACAGGATTCGACAAGCTC

                           160       170       180       190       200       210       220
CTR_ABC-B1_S.seq    CTATTGTCCATAGGCATCATTTCCGCAGTCGGAACAGGAGTGCTGCAGCCTATGAACACGATTCTGTTCGGAACT
CTR_ABC-B1_R.seq    CTATTGTCCATAGGCATCATTTCCGCAGTCGGAACAGGAGTGCTGCAGCCTATGAACACGATTCTGTTCGGAACT

                      230       240       250       260       270       280       290
CTR_ABC-B1_S.seq    CTAACTGGCGATATCATCAAGTATGCCGCATCGAAATTCAACCATAGCATGTCAGAAGACGACAGAATCAAAGCG
CTR_ABC-B1_R.seq    CTAACTGGCGATATCATCAAGTATGCCGCATCGAAATTCAACCATAGCATGTCAGAAGACGACAGAATCAAAGCG

                           310       320       330       340       350       360       370
CTR_ABC-B1_S.seq    GAAAATGATTTTTTCGATGGCGTCCAGTATTTCGCTATGATGAACTCGATTATCGCTGTGGGAATGGTCATCATC
CTR_ABC-B1_R.seq    GAAAATGATTTTTTCGATGGCGTCCAGTATTTCGCTATGATGAACTCGATTATCGCTGTGGGAATGGTCATCATC

                      380       390       400       410       420       430       440
CTR_ABC-B1_S.seq    AGCTATATATCGACGGTTACCTTCAACTACAGTGCGACGAGACAAGTATTCCGATTGAGGTCCACATATCTGAGT
CTR_ABC-B1_R.seq    AGCTATATATCGACGGTTACCTTCAACTACAGTGCGACGAGACAAGTATTCCGATTGAGGTCCACATATCTGAGT

                           460       470       480       490       500       510       520
CTR_ABC-B1_S.seq    AAAATACTCAATCAGGACATCACTTGGTATGACATGCACCAAACTGGAGATTTCTCGAGTAGAATGACAGAGGAT
CTR_ABC-B1_R.seq    AAAATACTCAATCAGGACATCACTTGGTATGACATGCACCAAACTGGAGATTTCTCGAGTAGAATGACAGAGGAT

                      530       540       550       560       570       580       590
CTR_ABC-B1_S.seq    CTGTTCAAATTCGAAGATGGAATTGGAGAAAAGGTCCCAATGTTCCTGAACCTTCAAATCGTTTTTTTCGTTTCA
CTR_ABC-B1_R.seq    CTGTTCAAATTCGAAGATGGAATTGGAGAAAAGGTCCCAATGTTCCTGAACCTTCAAATCGTTTTTTTCGTTTCA

                           610       620       630       640       650       660       670
CTR_ABC-B1_S.seq    TTGATAATAGCGCTAGTCAAAGGCTGGGAATTGGCACTCATCTGCCTCACCTCATTGCCAGCATCCCTCATCGCA
CTR_ABC-B1_R.seq    TTGATAATAGCGCTAGTCAAAGGCTGGGAATTGGCGCTCATCTGCCTCACCTCATTGCCAGCATCCCTAATCGCT

                      680       690       700       710       720       730       740
CTR_ABC-B1_S.seq    TTAGGGATCGTCGGTTTGTTAACCACCAAATTATCCAAAAAAGAACTGGATGCTTATGGTACTGCAGGTGCAATA
CTR_ABC-B1_R.seq    TTAGGGATTGTCGGTTTGTTGACCACCAAATTATCCAAAAAAGAACTGGATGCTTATGGTACTGCAGGTGCAATA

                           760       770       780       790       800       810       820
CTR_ABC-B1_S.seq    GCAGAAGAAGTTCTCTCTTCAATCAGGACAGTTATAGCCTTCGGCGGTCAACACAAAGAAATAGAGAGATACGGG
CTR_ABC-B1_R.seq    GCAGAAGAAGTTCTCTCTTCAATCAGGACAGTTATAGCCTTCGGCGGTCAACACAAAGAAATAGAGAGATACGGG

                      830       840       850       860       870       880       890
CTR_ABC-B1_S.seq    AACAATTTGATTTTCGCAAGGAAGAACAATATCAAAAGATCTCTGCTATCCGCGATAGGATTCGGTATCTTGTGG
CTR_ABC-B1_R.seq    AACAATTTGATTTTCGCAAGAAAGAACAATATCAAAAGATCTCTGCTATCCGCGATAGGATTCGGTATCTTGTGG

                           910       920       930       940       950       960       970
CTR_ABC-B1_S.seq    TTTCTGATTTATTCCAGTTACGCCTTAGCATTCTGGTACGGGGTCAAGCTGGTTCTGGAACAAAGGGATTGGGAG
CTR_ABC-B1_R.seq    TTTCTGATTTATTCCAGTTACGCCTTAGCATTCTGGTACGGGGTCAAGCTGGTTCTGGAACAAAGGGATTGGGAG

                      980       990      1000      1010      1020      1030      1040
CTR_ABC-B1_S.seq    AATCCTGTATACACTGCCGGTAACATGGTGACTGTATTCTTCAGTGTGATGAATGGTTCCATGAATTTCGGGATT
CTR_ABC-B1_R.seq    AATCCTGTATACACTGCCGGTAACATGGTGACTGTATTCTTTAGTGTGATGAATGGTTCCATGAATTTCGGGATT

                          1060      1070      1080      1090      1100      1110      1120
CTR_ABC-B1_S.seq    TCATCCCCGTACATCGAGGCATTCGGTATATCGAAGGCAGCAGCTTCTAAAATTTTCAGCGTTATCGACAATACC
CTR_ABC-B1_R.seq    TCATCCCCGTACATCGAGGCATTCGGTATATCGAAGGCAGCAGCTTCTAAAATTTTCAGCGTCATCGACAATACC

                     1130      1140      1150      1160      1170      1180      1190
CTR_ABC-B1_S.seq    CCCACCATCAATCTCTCGAAAGGAAAAGGGGAAATACTTGATACTCTCAAAGGGAATATCAAATTCAGGAATGTC
CTR_ABC-B1_R.seq    CCCACCATCAATCTCTCGAAAGGAAAAGGGGAAATACTTGATACTCTCAAAGGGAATATCAAATTCAGGAATGTC

                          1210      1220      1230      1240      1250      1260      1270
CTR_ABC-B1_S.seq    AATTTCCACTATCCATCCAGACCAGACGTTACGGTTTTACAAGACTTGAGTTTGGACATAAGAGCAGGCGATACC
CTR_ABC-B1_R.seq    AATTTCCACTATCCATCCAGACCAGACGTTACGGTTTTACAAGACTTGAGTTTGGACATAAGAGCAGGCGATACC

                     1280      1290      1300      1310      1320      1330      1340
```

```
CTR_ABC-B1_S.seq    GTAGCTCTGGTCGGAAGTTCCGGTTGTGGTAAATCAACTGTGATTCAACTGATCCAAAGATTTTATGACCCTGTC
CTR_ABC-B1_R.seq    GTAGCTCTGGTCGGAAGTTCCGGTTGTGGTAAATCAACTGTGATTCAACTGATCCAAAGATTTTATGACCCTGTC

                              1360      1370      1380      1390      1400      1410      1420
CTR_ABC-B1_S.seq    GCTGGCGAGGTGTCCATCGATGGTAAGAATATAAAAGACCTCGACCTCACCTGGATGCGAACGAACATCGGCGTC
CTR_ABC-B1_R.seq    GCTGGCGAGGTGTCCATCGATGGTAAGAATATAAAAGACCTCGACCTCACCTGGATGCGAACGAACATCGGCGTC

                         1430      1440      1450      1460      1470      1480      1490
CTR_ABC-B1_S.seq    GTGGGCCAAGAACCAGTTCTGTTCGGAACCACCATCATGGAAAATATAAAGTACGGAAATGCGGACGCAACCGAA
CTR_ABC-B1_R.seq    GTGGGCCAAGAACCAGTTCTGTTCGGAACCACCATCATGGAAAATATAAAGTACGGAAATGCGGACGCAACCGAA

                              1510      1520      1530      1540      1550      1560      1570
CTR_ABC-B1_S.seq    GATGACGTCGTCGTGGCAGCTAAGAAGGCGAATGCACATACTTTCATCAAATCGCTTCCGAACGGATACAACACT
CTR_ABC-B1_R.seq    GATGACGTCGTCGTGGCAGCTAAGAAGGCGAATGCACATACTTTCATCAAATCGCTTCCG----GATACAACACT

                         1580      1590      1600      1610      1620      1630      1640
CTR_ABC-B1_S.seq    CTGGTGGGCGAAAGGGGGGCGCAATTGTCTGGGGGGCAGAAGCAGCGAATAGCCATAGCCAGAGCTCTGGTGAGG
CTR_ABC-B1_R.seq    CTGGTGGGAGAAAGGGGGGCGCAATTGTCCGGGGGGCAGAAGCAGCGAATAGCCATAGCCAGAGCTCTGGTGAGG

                              1660      1670      1680      1690      1700      1710      1720
CTR_ABC-B1_S.seq    AAACCATCGATACTGCTCTTGGACGAAGCCACTTCCGCGTTGGACAATAACAGTGAGGCCAAAGTTCAAGCTGCT
CTR_ABC-B1_R.seq    AAACCATCGATACTGCTCTTGGACGAAGCCACTTCCGCGTTGGACAATAACAGTGAGGCCAAAGTTCAAGCTGCT

                         1730      1740      1750      1760      1770      1780      1790
CTR_ABC-B1_S.seq    CTAGACTCGGCTAGTGTGGATTGCACGACAGTCATTGTCGCTCATCGGTTGTCCACCATACAAGGAGCTAACAAG
CTR_ABC-B1_R.seq    CTAGACTCGGCTAGTGTGGATTGCACGACAGTCATTGTCGCTCATCGGTTGTCCACCATACAAGGAGCTAACAAG

                              1810      1820      1830      1840      1850      1860      1870
CTR_ABC-B1_S.seq    ATAATGGTGTTTTCGAAAGGCGCTGTTGTAGAACAAGGCACTCACGATGAGTTGATGGCACTTAAGAACGAGTAT
CTR_ABC-B1_R.seq    ATAATGGTGTTTTCGAAAGGCGCTGTTGTAGAACAAGGCACTCACGATGAGTTAATGGCACTTAAGAACGAGTAT

                         1880      1890      1900      1910      1920      1930      1940
CTR_ABC-B1_S.seq    TATAATCTAGTAACGACACAAGTCAAGAGTAAAGAAACGGTGACACAATATAGTAAGAGTGATAAGACTCAAGAA
CTR_ABC-B1_R.seq    TATAATCTAGTAACGACACAAGTCAAGAGTAAAGAAACGGTGACACAATATAGTAAGAGTTATAAGACTCAAGAA

                              1960      1970      1980      1990      2000      2010      2020
CTR_ABC-B1_S.seq    TATGATGATGATATCGATGAAGTTGTTCCAGTAGAAGCCTCCTTTGCAGCTGAAGATGATGAGGATGATTTCGTA
CTR_ABC-B1_R.seq    TATGATGATGATATCGATGAAGTTGTTCCAGTAGAAGCCTCCTTTGCAGCTGAAGATGATGAGGATGATTTCGTA

                         2030      2040      2050      2060      2070      2080      2090
CTR_ABC-B1_S.seq    TCAGACAGAAACATGAGATTGATTGATGTGATCAAAATGAATGCCCCAGAATGGCCACAAATTGTGGTGGCCAGT
CTR_ABC-B1_R.seq    TCAGACAGAAACATGAGATTGATTGATGTGATCAAAATGAATGCCCCAGAATGGCCACAAATTGTGGTGGCCAGT

                              2110      2120      2130      2140      2150      2160      2170
CTR_ABC-B1_S.seq    ATCGGGTCTACTGTTATAGGCTGTGCCATGCCAATTTTTTCTGTTTTATTTGGAAGCATTATTGGAACTCTAGCC
CTR_ABC-B1_R.seq    ATCGGGTCTACTGTTATAGGCTGTGCCATGCCAATTTTTTCTGTTTTATTTGGAAGCATTATTGGAACTCTAGCC

                         2180      2190      2200      2210      2220      2230      2240
CTR_ABC-B1_S.seq    AACAGTGATACAGAATACGTTAGAACAGAAACCAACAAATACGTCGTGTACTTTGTTATCGCAGGTGCTGTGGCT
CTR_ABC-B1_R.seq    AACAGTGATACAGAATACGTTAGAACAGAAACCAACAAATACGTCGTGTACTTTGTTATCGCAGGTGCTGTGGCT

                              2260      2270      2280      2290      2300      2310      2320
CTR_ABC-B1_S.seq    ATGGTATCAGTATTCCTTCAGATGTATATGTTTGGTATAGCTGGAGAAAAAATGACCGAGAGAATACGAGGCAAA
CTR_ABC-B1_R.seq    ATGGTATCAGTATTCCTTCAGATGTATATGTTTGGTATAGCTGGAGAAAAAATGACCGAGAGAATACGAGGCAAA

                         2330      2340      2350      2360      2370      2380      2390
CTR_ABC-B1_S.seq    ATGTTTTCAGCTATGCTGAACCAAGAAATCGGATTTTTCGACAAGAAAACCAACGGAGTTGGGGCTCTGTGTGCC
CTR_ABC-B1_R.seq    ATGTTTTCAGCTATGCTGAACCAAGAAATCGGATTTTTCGACAAGAAAACCAACGGAGTTGGGGCTCTGTGTGCC

                              2410      2420      2430      2440      2450      2460      2470
CTR_ABC-B1_S.seq    AAACTTTCTAGCGATGCTGCAAGCGTTCAGGGAGCAACAGGGCAAAGAGTTGGCGTAGTTTTGCAGTCGATGGCC
CTR_ABC-B1_R.seq    AAACTTTCTAGCGATGCTGCAAGCGTTCAGGGAGCAACAGGGCAAAGAGTTGGCGTAGTTTTGCAGTCGATGGCC

                         2480      2490      2500      2510      2520      2530      2540
CTR_ABC-B1_S.seq    ACATTTTGCCTAGCAGTTGGTCTCGCCATGTATTATGAATACAGATTGGGGCTTGTTACTGTGGCTTTCATGCCA
CTR_ABC-B1_R.seq    ACATTTTGCCTAGCAGTTGGTCTCGCCATGTATTATGAATACAGATTGGGGCTTGTTACTGTGGCTTTCATGCCA

                              2560      2570      2580      2590      2600      2610      2620
CTR_ABC-B1_S.seq    TTTCTTCTGATAGCATTCTTTTTCGAGAGGAGGAATTCTTCTGGACAGAATGATACACGAGACCAATCACTACAA
CTR_ABC-B1_R.seq    TTTCTTCTAATAGCATTCTTTTTCGAGAGGAGGAATTCTTCTGGACAGAATGATACACGAGACCAATCACTACAA
```

```
                            2630      2640      2650      2660      2670      2680      2690
CTR_ABC-B1_S.seq     AAATCAACGAAGATTGCCGTAGAAGGGGTAGGAAACATACGAACGGTAGCGTCTTTGGGTTTGGAGGAAAAGTTC
CTR_ABC-B1_R.seq     AAATCAACGAAGATTGCCGTAGAAGGGGTAGGAAACATACGAACGGTAGCGTCTTTGGGTTTGGAGGAAAAGTTC

                                 2710      2720      2730      2740      2750      2760      2770
CTR_ABC-B1_S.seq     CATCATCTGTATATATCCGAACTTCTACCACACTACAAAAATTCTAGTAGCGCTTCGTTACACTGGCGGGGAATA
CTR_ABC-B1_R.seq     CATCATCTGTATATATCCGAACTTCTACCACACTACAAAAATTCTAGTAGCGCTTCGTTACACTGGCGGGGAATA

                            2780      2790      2800      2810      2820      2830      2840
CTR_ABC-B1_S.seq     GTCTTTGGTTTGTCGAGGGGTTTGTCATTTTTCGCCTACTCCGCTGCCATGTACTACGGAGGCTATTTGATCAAA
CTR_ABC-B1_R.seq     GTCTTTGGTTTGTCGAGGGGTTTGTCATTTTTCGCCTACTCCGCTGCCATGTACTACGGAGGCTATTTGATCAAA

                                 2860      2870      2880      2890      2900      2910      2920
CTR_ABC-B1_S.seq     AACGAAAACTTATCGTACGAGAAAGTTTTCAAAGTTTCCCAAGCGCTCATAATGGGAACTACTTCCATAGCCAAT
CTR_ABC-B1_R.seq     AACGAAAACTTATCGTACGAGAAAGTATTCAAAGTTTCCCAAGCGCTCATAATGGGAACTACTTCCATAGCCAAT

                            2930      2940      2950      2960      2970      2980      2990
CTR_ABC-B1_S.seq     GCACTCGCTTTCACTCCAAACTTCACGAAAGGCTTGAATGCAGCGAAGAGCGTTCAGAAGTTCCTGGAGAGGATG
CTR_ABC-B1_R.seq     GCACTCGCTTTCACTCCAAACTTCACGAAAGGCTTGAATGCAGCGAAGAGCGTTCAGAAGTTCCTGGAGAGGATG

                                 3010      3020      3030      3040      3050      3060      3070
CTR_ABC-B1_S.seq     CCGAAAATCAGGGACGATATGAATTCCAAAGATGTGAATGAGGTGGAAGGTGACATCTCATTTGCGAAAATCAAA
CTR_ABC-B1_R.seq     CCAAAAATCAGGGACGATATGAATTCCAAAGATGTGAATGAGGTGGAAGGTGACATCTCATTTGCGAAAATCAAA

                            3080      3090      3100      3110      3120      3130      3140
CTR_ABC-B1_S.seq     TTCGCATATCCAACCAGACCAGGCACAACGGTGCTTCGTGACCTTGACCTTAGGATATTCAAGGGGAAAACGGTG
CTR_ABC-B1_R.seq     TTCGCATATCCAACCAGACCAGGTACAACGGTGCTTCGTGACCTTGACCTTAGGATATTCAAGGGGAAAACGGTG

                                 3160      3170      3180      3190      3200      3210      3220
CTR_ABC-B1_S.seq     GCGCTCGTAGGGCAGAGTGGCTGCGGCAAATCTACCCTCATACAGCTCATCGAGCGATTCTACGATCCAACCGGA
CTR_ABC-B1_R.seq     GCGCTCGTAGGGCAGAGTGGCTGCGGCAAATCTACCCTCATACAGCTCATCGAGCGATTCTACGATCCAACCGAA

                            3230      3240      3250      3260      3270      3280      3290
CTR_ABC-B1_S.seq     GGAGAAGTGATGCTGGACGACATAGACGTCAAAAGAATGAAGCTGCGTTCTCTGAGATCGCATCTGGGGATCGTG
CTR_ABC-B1_R.seq     GGAGAAGTGATGCTGGACGACATAAACGTGAAAAGAATGAAGCTGCGTTCTCTGAGATCGCATCTGGGGATCGTG

                                 3310      3320      3330      3340      3350      3360      3370
CTR_ABC-B1_S.seq     TCGCAGGAACCCAACCTCTTCAACAAGACTATCAGGGAGAACATTTCGTATGGGGACAATGGACGTGTTGTTCAG
CTR_ABC-B1_R.seq     TCGCAGGAACCCAACCTCTTCAACAAGACTATCAGGGAGAACATTTCGTATGGGGACAATGGACGTGTTGTTCAA

                            3380      3390      3400      3410      3420      3430      3440
CTR_ABC-B1_S.seq     ATGGATGAGGTTATACAGGCTGCCGTGAATGCTAACATCCACACCTTCATCAGCGGACTACCAAAGGGTTATGAA
CTR_ABC-B1_R.seq     ATGGATGAGGTTATACAGGCTGCCGTGAATGCTAATATCCACACCTTCATCAGCGGACTACCAAAGGGTTATGAA

                                 3460      3470      3480      3490      3500      3510      3520
CTR_ABC-B1_S.seq     ACTACGCTGGGAGAAAAAGCTGTGCAATTATCAGGTGGACAGAAACAGCGAATCGCTATTGCTAGGGCCTTAGTA
CTR_ABC-B1_R.seq     ACTACGCTGGGAGAAAAAGCTGTGCAATTATCAGGTGGACAGAAACAGCGAATCGCTATTGCTAGGGCCTTAGTA

                            3530      3540      3550      3560      3570      3580      3590
CTR_ABC-B1_S.seq     AGAAATCCGAAAGTATTATTACTGGATGAGGCTACCTCTGCGTTGGATACTGAAAGCGAAAAGGTTGTTCAAGAA
CTR_ABC-B1_R.seq     AGAAATCCGAAAGTATTATTACTGGATGAGGCTACCTCTGCGTTGGATACTGAAAGCGAAAAGATTGTTCAAGAA

                                 3610      3620      3630      3640      3650      3660      3670
CTR_ABC-B1_S.seq     GCTCTAGACCAAGCCAAGTTGGGTAGAACGTGTATAACAATCGCTCATCGACTTTCGACCATCCAAGATGCAGAT
CTR_ABC-B1_R.seq     GCTCTAGACCAAGCCAAGATGGGTAGAACGTGTATAACAATCGCTCATCGACTTTCGACCATCCAAGATGCAGAT

                            3680      3690      3700      3710      3720      3730      3740
CTR_ABC-B1_S.seq     ATGATTTGTGTGATTGACCGGGGAATAGTAGCCGAAGCTGGAACTCATGCAGAGCTATTAGAGAAAAAAGGTCTC
CTR_ABC-B1_R.seq     ATGATTTGTGTGATTGACCGGGGAATAGTAGCCGAAGCTGGAACTCATGCAGAGCTATTAGAGAAAAAAGGTCTC

                                 3760      3770
CTR_ABC-B1_S.seq     TATTATAAGTTGCAAAGACAGACAACTTGA
CTR_ABC-B1_R.seq     TATTATAAGTTGCAAAGACAGACAACTTGA
```

Figure S1. Comparison between CtABCB1 cDNA sequences derived from either the susceptible or the resistant populations. Nucleotide sequences corresponding to the cDNAs (open reading frames) of CtABCB1 cloned from the *C. tremula* susceptible (S) and the resistant (R) populations were aligned using CLUSTAL W (version 2.0.12). The 23 single nucleotide polymorphisms (SNPs) are shaded in grey. Only 5 SNPs are non-synonymous. The four-base pair deletion present in *abc-b*1 is located at position 1561.

CTR_ABC-B1	MGKREKYSLDKKNKSPLDVEFTKNEEKEGDKTKQVSFFQMFRYATGFDK**LLLSIGIISAVGTGVLQPM**	68
	Y (above N)	
CTR_ABC-B1	NTILFGTLTGDIIKYAASKFNHSMSEDDRIKAENDFFDGVQ**YFAMMNSIIAVGMVIISYISTV**TFNYS	136
CTR_ABC-B1	ATRQVFRLRSTYLSKILNQDITWYDMHQTGDFSSRMTEDLFKFEDGIGEKVP**MFLNLQIVFFVSLIIA**	204
CTR_ABC-B1	**LVKGW**ELA**LICLTSLPASLIALGIVGLLTT**KLSKKELDAYGTAGAIAEEVLSSIRTVIAFGGQHKEIE	272
CTR_ABC-B1	RYGNNLIFARKNNIKR**SLLSAIGFGILWFLIYSSYALAFWY**GVKLVLEQRDWENP**VYTAGNMVTVFFS**	340
CTR_ABC-B1	**VMNGSM**NFGISSPYIEAFGISKAAASKIFSVIDNTPTINLSKGKGEILDTLKGNIKFRNVNFHYPSRP	408
CTR_ABC-B1	DVTVLQDLSLDIRAGDTVALV**GSSGCGKSTV**IQLIQRFYDPVAGEVSIDGKNIKDLDLTWMRTNIGVV	476
	4 bp deletion (arrow between P and N)	
CTR_ABC-B1	GQEPVLFGTTIMENIKYGNADATEDDVVVAAKKANAHTFIKSLPNGYNTLVGERGAQ**LSGGQKQRIAI**	544
CTR_ABC-B1	**ARAL**VRKPSILLLDEATSALDNNSEAKVQAALDSASVDCTTVIVAHRLSTIQGANKIMVFSKGAVVEQ	612
CTR_ABC-B1	GTHDELMALKNEYYNLVTTQVKSKETVTQYSKSDKTQEYDDDIDEVVPVEASFAAEDDEDDFVSDRNM	680
CTR_ABC-B1	RLIDVIKMNAPEWPQ**IVVASIGSTVIGCAMPIFSVLFGSII**GTLANSDTEYVRTETNKYV**VYFVIAGA**	748
CTR_ABC-B1	**VAMVSVFLQM**YMFGIAGEKMTERIRGKMFSAMLNQEIGFFDKKTNGVGALCAKLSSDAASVQGATGQR	816
CTR_ABC-B1	**VGVVLQSMATFCLAVGLAMYY**EYR**LGLVTVAFMPFLLIAFFFE**RRNSSGQNDTRDQSLQKSTKIAVEG	884
CTR_ABC-B1	VGNIRTVASLGLEEKFHHLYISELLPHYKNSSSASLHWR**GIVFGLSRGLSFFAYSAAMYYGGY**LIKNE	952
	Y (above N)	
CTR_ABC-B1	NLSYEK**VFKVSQALIMGTTSIANALAFTP**NFTKGLNAAKSVQKFLERMPKIRDDMNSKDVNEVEGDIS	1020
CTR_ABC-B1	FAKIKFAYPTRPGTTVLRDLDLRIFKGKTVALV**GQSGCGKSTLI**QLIERFYDPTGGEVMLDDIDVKRM	1088
CTR_ABC-B1	KLRSLRSHLGIVSQEPNLFNKTIRENISYGDNGRVVQMDEVIQAAVNANIHTFISGLPKGYETTLGEK	1156
CTR_ABC-B1	AVQ**LSGGQKQRIAIARAL**VRNPKVLLLDEATSALDTESEKVVQEALDQAKLGRTCITIAHRLSTIQDA	1224
CTR_ABC-B1	DMICVIDRGIVAEAGTHAELLEKKGLYYKLQRQTT	1259

Figure S2. Predicted protein sequence of CtABCB1. Feature predictions include 12 transmembrane helices (pink), two ATP-binding domains (green), two transporter motifs (blue), and predicted glycosylation sites on the outside loops (underlined in blue, and indicated by 'Y'). The position of the four-base-pair deletion is indicated by a red arrow.

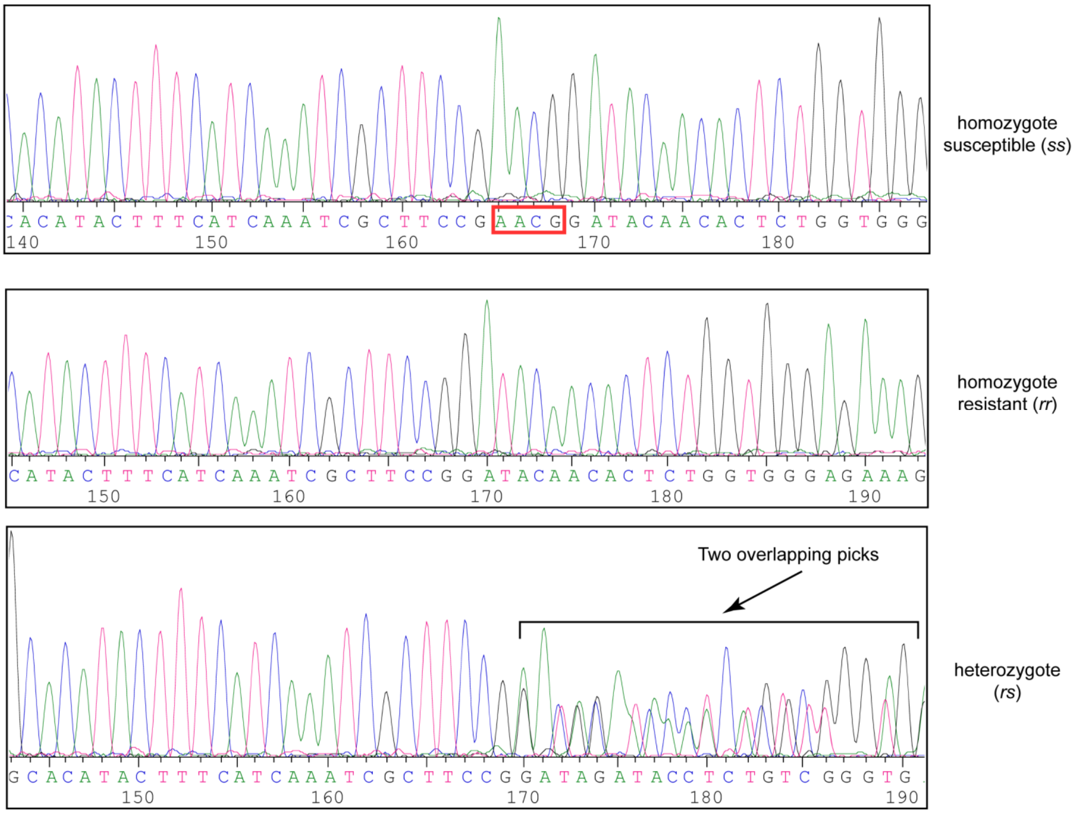

Figure S3. Determination of the genotype for the backcrosses between susceptible and resistant *C. tremula*. Genomic DNA was prepared from the grandparents, parents and offspring of each backcross. A primer pair was designed to amplify by PCR the region where the deletion is located in CtABCB1. PCR products were then processed by Sanger sequencing. The four nucleotides present in the "normal" version of *abc-b*1 but absent in resistant individuals is boxed in red.

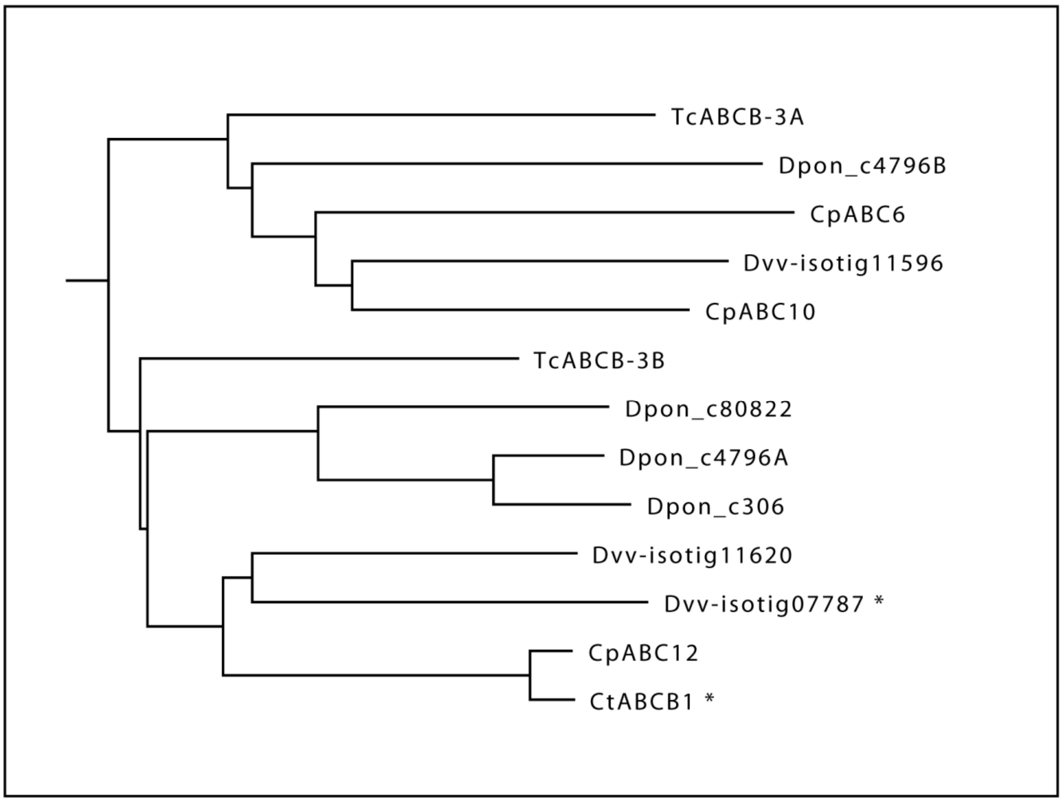

Figure S4. Neighbor-joining tree of full-transporter ABCB protein sequences from Coleoptera. Tc: *Tribolium castaneum*, Dpon: *Dendroctonus ponderosae*, Cp: *Chrysomela populi*, Dvv: *Diabrotica virgifera virgifera*, Ct: *Chrysomela tremula*. * The asterisks denote proteins genetically linked to Cry3 toxin resistance.

```
TcABCB-3A          1 MAKDDFQENGLEETVPLNTEYVKNKLKIKKTKDGKKEDKKEEK-------   43
Dpon_c4796B        1                          MEIKKNHSSDQQEPPERQ-------   18
CpABC6             1                          MCQVSYRQPESLARSLKC-------   18
Dvv-isotig11596    1                          MGNKKNENDLKCEKNKTN-------   18
CpABC10            1                          MGERKLYSVDKSEKKDKSKKVPNDS   25
TcABCB-3B          1              MGKKAYDLESSEKNGKKDALTPEFVQEPSEK-----L   32
Dpon_c80822        1              MSDEK---HDQKNGVDKPSPLEAKFQPPPDP-----D   29
Dpon_c4796A        1              MAAKKTDGDQDGKAADKKQALDVKFQSIDDPADP--D   35
Dpon_c306          1              MTER-----HDGTATDKERASDVNVPPIDDP------   26
Dvv-isotig11620    1              MTEEK----KHSIKDKEKIGIDAQFVNSEEPK-----   28
Dvv-isotig07787    1              MG-TK----KDDIKEKKTNALDAKFVKKKENE-----   27
CpABC12            1              MGKRE----KYSLDKKNKSPLDVEFTKNEEKEG----   29
Ct-ABCB1           1              MGKRE----KYSLDKKNKSPLDVEFTKNEEKEG----   29

TcABCB-3A         44 ----KISYFQLFRYTTLQDKLCIALGTLCAVICGCIQPYVMILFGDVTEV   89
Dpon_c4796B       19 ---PDVPYHQLFQFATLLDKLLIAVGIIASIICGVFQPYLMVLFGDVSGV   65
CpABC6            19 ----IYFYFKCFRYATALDKICLLVASSCTICCGILMPFAMLFFGDVTGS   64
Dvv-isotig11596   19 ----EISYLRLFQYCTTLDIIFMSIGAICAALSGIVQPYSMTLFGDVTGA   64
CpABC10           26 KEKQTIPYYRLFQYSTNLDKICLLLASACTILCGALQPFMMTIFGEITGS   75
TcABCB-3B         33 EKAPPVGFFKLFRYATKWDTFLMIIAVFASIGTGILQPLNTLLFGDLTGT   82
Dpon_c80822       30 EDISPPSFLQLYRYATGWEKFLLFIGVISALASGAIQPLNNLLFGQLIQT   79
Dpon_c4796A       36 EAVDASSFTGLYRFATQPQKYVIVIGAIIALISGALQPLNNLLFGDLTQT   85
Dpon_c306         27 ESTDASSFTGLYRFATRPQKCVIIIGAIIALICGALQPLNHLLFGDLTQI   76
Dvv-isotig11620   29 EKIKNVSFPQMFRYASTYDKFLMVVGLISATGTGVLQPLNTILFGSLTGD   78
Dvv-isotig07787   28 TKIKPISFFGMFKYASKGDKLLMFIGTLSAVITGCLPPINTILFGELAGN   77
CpABC12           30 DQTKQVSFFQMFRYATGLDKVLLSIGIISAFGTGVLQPMNTILFGTLTGD   79
Ct-ABCB1          30 DKTKQVSFFQMFRYATGFDKLLLSIGIISAVGTGVLQPMNTILFGTLTGD   79
                            .   .....      . .    .  .*   *     ** .

TcABCB-3A         90 IIQFAETLKSN-NSEINRTQAVDDLFRGVTDFAIYSSSSGIVMIITTYLA  138
Dpon_c4796B       66 LLDFTTAMNAN-LTFEEEKLATEKLYDGTEYFAIMTSVSALIILVCTYIS  114
CpABC6            65 IIDYAAAIKES-PPEDEKEKLIETLRHEIGLFAAQAAIIIIAVIALLYLS  113
Dvv-isotig11596   65 IVTYASNYNES-LSEPEKTLLADELINAVWLFGMKSVGVGIGVILTTYIS  113
CpABC10           76 IVDYATAFNDS-ITEEQKAVLVEQLWSDMKIFVIYSCIIGIVTILATYIS  124
TcABCB-3B         83 IVDYVFTINSNETSEEQKQNATDVFIDGITDFAVYNTLIGVGMLVLSYIS  132
Dpon_c80822       80 IVDYAQICYDD-PSSDQCVSAGDGLIDGVTHYAIWNSAIGVGIFVTSYVS  128
Dpon_c4796A       86 IIEYSQACFI--PSSTECGAAGDNLLAGIKHFALWNSLNGVAIMITAYLA  133
Dpon_c306         77 IIEYSEACLN--PNSTNCSVAGDNLSAGIKHFGIWSSINGVAIMITGYLA  124
Dvv-isotig11620   79 IIAYATS-IQINLPADQKKIAEDNFFDGIRYFALMNSLIGIGMFVFSYLA  127
Dvv-isotig07787   78 AVQYAETLYNATLSQNEQAEAQEKFFDGIKFFALANSMVGVAMLCFSYLS  127
CpABC12           80 IIKYAASKFNDSMSEDDRIKAENDFFDGVQYFALMNSIIAVGMVIISYIS  129
Ct-ABCB1          80 IIKYAASKFNHSMSEDDRIKAENDFFDGVQYFAMMNSIIAVGMVIISYIS  129
                      . .                           .        .      *..

TcABCB-3A        139 GILFSSSALRQIFHIRKLILQKTLNMDISWYDLNKTGDFATTFTENLSKL  188
Dpon_c4796B      115 VVFFTQSSLRQTCKMRKLFMEKTINQDIGWYDQNQTGDFASIITDNIPKI  164
CpABC6           114 VVLFSYTAVRQIFKMRTSFLENILNQDIEWFDRNHSGDFTTIFTQNISKI  163
Dvv-isotig11596  114 TVLFIYSASRQIFKIRKAFLEKTLNQDIAWFDQNRTGDFASTFTQNISKL  163
CpABC10          125 VVLYSFSANRQIFKMRKMFLEKTLHQDIGWFDINQTGDFATTFTQNISKI  174
TcABCB-3B        133 TEFFNYTALKQVFKVRTLYLEKVFNQDISWYDVNNTGDFSSRMSDDLSKF  182
Dpon_c80822      129 TEAFSYTAIKQVFRVRSKYLQALLNKDVPWFDVHNSGDFASRMTDDLQRY  178
Dpon_c4796A      134 TEAFSYNAIKQVFKVRSLYLQRLLNKDIPWFDVHNSGDFSSRMADDLSKF  183
Dpon_c306        125 TEAFSYNAIKQVFRVRSLYLQKLLNKDISWFDVHNSGDFSSRMADDLSKF  174
Dvv-isotig11620  128 TVTFNYSAMRQIFKIRSAYLKSILNQDVGWYDINQTGDFASRMSDDLFKF  177
Dvv-isotig07787  128 TITFNYSSTKQIFQIRSDYLKSILNQDISWYDQNQTGDFASRMSDDLSKF  177
CpABC12          130 TVTFNYSATRQVFRLRSIYLSKILNQDITWYDMHQTGDFSSRMTEDLFKF  179
Ct-ABCB1         130 TVTFNYSATRQVFRLRSTYLSKILNQDITWYDMHQTGDFSSRMTEDLFKF  179
                        .   . .*  ..*   .    . *. *.* . .***..  .  . .
```

```
TcABCB-3A        189 EEGIGEKVGIFLYFETIFVTGIVMGLVLGWELALICLISLPVSFAVAFLI  238
Dpon_c4796B      165 EDGIGEKVGVVVFLGTTCVSGMIWALIKGWQLALVCLASLPLQTLVMGAI  214
CpABC6           164 EDGIGEKIGLFLYSIAWFLTGIIISLYEGWKLALVAMVSLPLSTILILII  213
Dvv-isotig11596  164 EEGIGEKIGTFLFFESTFVAGCVLGLVKGWKLALVCMVSLPLSTTIMTII  213
CpABC10          175 EEGIGEKIGVFLFFESTFVAGIIIALIKGWKLALVCVVSLPLSTIIMALI  224
TcABCB-3B        183 EDGIGEKVPMFVHFQATFLASLIMALVKGWQLALICLVSLPLSMIAIGII  232
Dpon_c80822      179 EDGIGEKMPLFLMLQGTFISAITLAMVKGWQLALICLISLPVSLLALGII  228
Dpon_c4796A      184 EDGIGEKVPLFLTLQGSFISAITLALVKGWELALICLISLPVSLIAVGLI  233
Dpon_c306        175 EDGIGEKVPLFLTLQGSFISAITLALVKGWELALICLISLPVSFIAVGLI  224
Dvv-isotig11620  178 EDGIGEKVPVFWSFQVVFLTSLIIALVKGWELALICLTSLPATLITIGIV  227
Dvv-isotig07787  178 EDGIGEKVPMFIHFQVLFVAAVGTALAKGWELALISLVAIPASLVSFGVI  227
CpABC12          180 EDGIGEKVPMFLNLQIVFFVSLIIALVKGWELALICLTSLPASLIALGIV  229
Ct-ABCB1         180 EDGIGEKVPMFLNLQIVFFVSLIIALVKGWELALICLTSLPASLIALGIV  229
                     *.*****.                 .  ** ***... ..*        .

TcABCB-3A        239 SWLSTKFSKQELEAYANAGAIAEEVLSSVRTVVAFDGQGKEFERYEKHLQ  288
Dpon_c4796B      215 AWFSAKYCKQEMAAYSAAGAIAEEVLSSIKTVVAFDGQEKEEKRYEKFVK  264
CpABC6           214 TKIAAKFSKGEMEAYQDAGAIAQEVFGSIKTVVAFNGQDKECKIYEDHLV  263
Dvv-isotig11596  214 SWISTKFSAQEMESYGEAGTIAEEVFTAIKTVVAFDGQDKEIERYNKHLV  263
CpABC10          225 TTISTKFSQQELEAYGLAGSVAQEVLGSIRTVVAFEGQQKESSRYHQHLV  274
TcABCB-3B        233 AVLTSKLAKKEQDAYGSAGSIAEEVLTSIRTVIAFGGQHKEITRYDEELE  282
Dpon_c80822      229 AFLTTKFTSKELDAYGTAGSLAEEVFTFIRTVVAFGGEKQEKERYDESLV  278
Dpon_c4796A      234 AFLTTKFSKKELDAYAEAGSIAEEVLTSIRTVIGFGGEKLETQRYDKSLI  283
Dpon_c306        225 AYLTTQFSKKELDAYAEAGSIAEEVITSIRTVIGFGGEKLETQRYEKNLI  274
Dvv-isotig11620  228 GLLTTKLAKNELEAYGAAGSIAEEALSLIRTITAFGGQKKEVDRYNKNLV  277
Dvv-isotig07787  228 NLLTTKLAKNEMEAYAASGSIAEEVFSLIRTVTAFGGQQKEIDRYKEHLV  277
CpABC12          230 GLLTTKLSKKELDAYGTAGAIAEEVLSSIRTVIAFGGQHKEIERYGNNLI  279
Ct-ABCB1         230 GLLTTKLSKKELDAYGTAGAIAEEVLSSIRTVIAFGGQHKEIERYGNNLI  279
                        ... .  *  .*  .*..*.*    ..*.  * *.  *   *   .

TcABCB-3A        289 AAKKNNIRKNLFTGVSNAVMWFFVFASYALSFWYGVGLILKEKELPYEER  338
Dpon_c4796B      265 IAQNNNVKRCLFNSINQGFLWFLAYGCYALAFWYGVGLVIEERNLPEADR  314
CpABC6           264 HAERNNVLRCIFNSTNNFFLWFSMFACYSLTYWCGIHLIIGDRDLPSEER  313
Dvv-isotig11596  264 DAKKNNLKRSFFTAVSNGCLWFFVYACYALSFWYGVTLILNDRHLPEHEQ  313
CpABC10          275 HAQNNNIKRGLFNALSNGCLWFFVYACYALSFWYGVGLILEERQLPEAER  324
TcABCB-3B        283 FAKKNNIKRQSMTAIGFGLLWFFIYGSYALAFWYGVKLVLEDRNKPAKDK  332
Dpon_c80822      279 FARNNNIRRHFFEGIGYGTLWFCVFSSYALAFWYGVKLMLDG------NP  322
Dpon_c4796A      284 FARNNNIRRHFFEGIGYGLLWLCIFSSYGLAFWYGVKLMLDG------NP  327
Dpon_c306        275 FAKDNNIRRHFFEGIGYGMLWLCIFSSYGLAFWYGIKLMLDG------NP  318
Dvv-isotig11620  278 EAKNNNIRRSMFSALGFGLLWFMIYASYALAFWYGVKLVLRDRT--ATNQ  325
Dvv-isotig07787  278 SARKNNIKRTMIVGIGFGLVYLLLYSSYALSLWYGVKLVLKDRF--NPNA  325
CpABC12          280 FARKNNIKRSLLSAIGFGILWFLIYSSYALAFWYGVKLVLEQRD--WDNP  327
Ct-ABCB1         280 FARKNNIKRSLLSAIGFGILWFLIYSSYALAFWYGVKLVLEQRD--WENP  327
                      *  **. .          .    . .* *. * *. *..

TcABCB-3A        339 VYTPGNMVSVFFCTLMASWNFGTGAPYFEIFGTACGAAAKVFEILDTKPD  388
Dpon_c4796B      315 VYTPGNMMGVFFATLIASWNCGTISPYLEVFGMARGAAFKVFQVLESRPE  364
CpABC6           314 VYTPSSLVTIFFATLGATWYFGLAAPLLEVFATARTAAQKVFFILDSKPK  363
Dvv-isotig11596  314 VYTPANMVSVFFSTLIATWNFGLGAPLLETFGTAKGAAQKIFFVLDSKPV  363
CpABC10          325 VYTAGNMVSVFFCTLVATWNFGMGAPILEIFGAAKGAAQKIFAVLDSEPK  374
TcABCB-3B        333 VYDPGTMVTVFFSVMTGSMNFGISSPYIEAFGVARAAASKVYQIIDNIPK  382
Dpon_c80822      323 VYTPGNMITVFFSVMAGSVGFGMASPFIEAFSTAKAAGGKIFHIIETSPT  372
Dpon_c4796A      328 VYTPGNMITVFFSVMTGSMNFGVASPFIEAFATAKAAGGKIFHIIDTSPT  377
Dpon_c306        319 VYTPGNMLTV---VMAGSMSFGSASPFIEAFATAKAAGRKIFHMIKTSPI  365
Dvv-isotig11620  326 IYTPSNMVTVFFSVMTGSMNFGIASPYIEAFGISKAAASKIFSVIDHKPT  375
Dvv-isotig07787  326 SYTPANMVTIFFAVLSASLNFGAASTYLEMFAISKAAAAKIFNVIESKPD  375
CpABC12          328 VYTAGNMVTVFFSVMNGSMNFGISSPYIEAFGISKAAASKIFSVIDNTPT  377
Ct-ABCB1         328 VYTAGNMVTVFFSVMNGSMNFGISSPYIEAFGISKAAASKIFSVIDNTPT  377
                      *    .. .    .  .   *  .   * *  .  *  *.. ..   *
```

```
TcABCB-3A        389 INLSKTKGLKPKNLKGDIVFKDVSFHYPSRPDVKILQNFSIEIKAGQTVA  438
Dpon_c4796B      365 MYKQYDVGKRP-DFMSNIAFKNVKFSYPSRANVQVLKNINLEIRFGETVA  413
CpABC6           364 INTIKEKGRKLYRFQNCIRFDEVYFSYPTRSELKVLWGFDLQINIGETVA  413
Dvv-isotig11596  364 IRKFANSGIVINDAALEVTFEDVHFSYPSRSDVKILRGINLTISKGETVA  413
CpABC10          375 INQSKSVGKKLDDFKSNIRFENVFFSYPARPDVKILQGLNLQINFGETIA  424
TcABCB-3B        383 INLSKGNGDKIDNLKGDIKFRNVRFVYPSRQDVPILLGLDLDIKAGQTVA  432
Dpon_c80822      373 INLSKNNGKKPEEVKGNISFKNVHFYYPSRKEVEVLQGLTLDIAAGDTVA  422
Dpon_c4796A      378 INQSKNSGAKLDQVRGNIKLQNVKFQYPSRKDVPILQGIDLEIQAGDTVA  427
Dpon_c306        366 INQSKNNGAKLAQVRGNIKLQNVKFQYPSRKDVPVLQGIDLEIQAGDTVA  415
Dvv-isotig11620  376 INLSKGNGKTLNVLIGNIQFKDVAFRYPSRKDVPILKGLSLNIKSGDTVA  425
Dvv-isotig07787  376 INLSKSNGAKLDHLKGNIQFKNVKFHYPSRPDIPVLQGLDIEINPGETVA  425
CpABC12          378 INLSKGKGQQLDALKGNIKFRNVNFHYPSRPDVTVLQDLSLDIKAGDTVA  427
Ct-ABCB1         378 INLSKGKGEILDTLKGNIKFRNVNFHYPSRPDVTVLQDLSLDIRAGDTVA  427
                     .      *         .    * * **.*  . .*    . *  * *.*

TcABCB-3A        439 LVGSSGCGKSTCIQLIQRFYDAVTGTVKIDDNNIKDLNLTWLRSKIGVVG  488
Dpon_c4796B      414 LVGPSGSGKSTIVQLMQRFYDPNSGMISIDDVNLKDVNLSYLRQNVGVVS  463
CpABC6           414 LVGSSGCGKSTCIQLLQRFYDPTLGRITIDDVDIKDMSLKWLREKIAVVS  463
Dvv-isotig11596  414 LVGKSGCGKSTCIQLLQRFYDPDMGQIKINGIDIKQLNLDWLKQKISVVS  463
CpABC10          425 LVGSSGCGKSTCIQLLQRFYDPVVGNVLIDGVNLRDLNLTWLRQKIAVVS  474
TcABCB-3B        433 LVGSSGCGKSTCIQLIQRFYDPLEGEVSLDGKNLKDFDLTWLRNNIGVVG  482
Dpon_c80822      423 LVGSSGCGKSTVLQLVQRFYDALEGRVTIDGQDVKELDLTSYRQNIGVVS  472
Dpon_c4796A      428 LVGSSGCGKSTVLQLVQRFYDPLQGQVFIDGKDVKDLDLSWYRQSIGVVS  477
Dpon_c306        416 LVGRSGCGKSTLLQLVQRFYDPLQGQVFIDEKDVKELDLSWYRQYIGVVS  465
Dvv-isotig11620  426 LVGSSGCGKSTVIQLLQRLYDADSGEVTIDGKNIKEYDLTWLRSQIGVVG  475
Dvv-isotig07787  426 LVGSSGCGKSTVVQLIQRFYNAVSGEILVDGTNIQNLDLTWYRNNIGVVG  475
CpABC12          428 LVGSSGCGKSTVIQLIQRFYDPSAGEVSIDGKNIRDLDLTWMRTNIGVVG  477
Ct-ABCB1         428 LVGSSGCGKSTVIQLIQRFYDPVAGEVSIDGKNIKDLDLTWMRTNIGVVG  477
                     *** **.**** .**.** *    * . .    ..   *   .  . **

TcABCB-3A        489 QEPALFGATIAENIKFGNVTATQSDVERAAKKANAHNFIQKLPRGYNTVV  538
Dpon_c4796B      464 QEPSLFATTIAENIRYGKLSATMEEIIAAAKKANAHRFVTNLPFGYQTVI  513
CpABC6           464 QEPALFTTTVAENIRFGKEGAPQEEIETAARKARVHEFILTLPNGYETVI  513
Dvv-isotig11596  464 QEPDLFSTTIAENIRYGKLNATQQEIENAAKKADIHTFIQTLPRGYQTVL  513
CpABC10          475 QEPALFATTIAENIRLGKLDAKQAEIEEAAKKANVHKFILTLPHGYDTVI  524
TcABCB-3B        483 QEPVLFATTIAENIRYGNSKATDEEIKNAAIKANAHEFIKKLPSGYDTLV  532
Dpon_c80822      473 QEPVLFGTTIYENIRYGNKDATEEDITRAAKMANAHQFIKGLPHGYYTLV  522
Dpon_c4796A      478 QEPVLFGTTIAENIRYGNKEATEQDIVQAAKRANAHRFIKGLPEGYHTLV  527
Dpon_c306        466 QEPVLFGTTIAENIKYGNRDATELDIVQAAKMANAHQFIKGLPGGYHTLV  515
Dvv-isotig11620  476 QEPILFGTSILENIRYGKDGVTEEDVIQAAKKANAHNFIKALPNGYNTLV  525
Dvv-isotig07787  476 QEPVLFGTTILENIRYGKEEATEEEIIEAAKKANAHAFIKLLPKGYNTLV  525
CRW424                                                    FIKLLPKGYNTLV
CpABC12          478 QEPVLFGTTIMENIKYGNAEANEDDVIAAAKKANAHTFIKSLPNGYNTLV  527
Ct-ABCB1         478 QEPVLFGTTIMENIKYGNADATEDDVVVAAKKANAHTFIKSLPNGYNTLV  527
                     *** ** ... ***. *       ..  **  *  * *.  ** ** *..

TcABCB-3A        539 GERGAQLSGGQKQRIAIARALIREPKILLLDEATSALDTTSEAEVQAALD  588
Dpon_c4796B      514 GERGSQLSGGQKQRIAIARALIKAPNLLILDEATSALDTASEVEVQQALD  563
CpABC6           514 GERGGQLSGGQKQKLAIARALIRRPQILLLDEATSALDTSSESEVQAALD  563
Dvv-isotig11596  514 GERGTQLSGGQKQKIAIARALVRTPEFLLLDEATSALDTTSEAEIQEALD  563
CpABC10          525 GERGAQLSGGQKQRIAIARALVRKPEILLLDEATSALDTTSEAEVQAALD  574
TcABCB-3B        533 GERGAQLSGGQKQRIAIARALVRNPAILLLDEATSALDTNSEAKVQAALD  582
Dpon_c80822      523 GERGAQLSGGQKQRIAIARALVRNPAILLLDEATSALDTNSEAKVQAALD  572
Dpon_c4796A      528 GERGAQLSGGQKQRIAIARALVRNPAILLLDEATSALDTNSEAKVQAALD  577
Dpon_c306        516 GERGAQLSGGQKQRIAIARALVRNPAILLLDEATSALDTNSEAKVQAALD  565
Dvv-isotig11620  526 GEKGAQLSGGQKQRIAIARALVRNPTLLLLDEATSALDNTSEAKVQAALD  575
Dvv-isotig07787  526 GERGTQLSGGQKQRIAIARALVRKPTLLLLDEATSALDNASEAKVQEALD  575
CRW424               GERGTQLSGGQKQRIAIARALVRKPTLLLLD
CpABC12          528 GERGAQLSGGQKQRIAIARALVRKPTILLLDEATSALDNNSEAKVQAALD  577
Ct-ABCB1         528 GERGAQLSGGQKQRIAIARALVRKPSILLLDEATSALDNNSEAKVQAALD  577
                     **.* ********..******.. *  *.*********. **  .* ***
```

```
TcABCB-3A          589 AVSGECTTIIVAHRLSTIRNANRIVVVSHGSVIEEGTHSELMAKKGAYFD  638
Dpon_c4796B        564 AISGECTKLVVAHRLSTIRNATRIIVFDQGEVVEHGSHAQLMAAKGVYYN  613
CpABC6             564 SISGECTTIIVAHRLSTIRNAKKIVYISEGRVSEIGSHAELMAKKGAYFK  613
Dvv-isotig11596    564 SIRGTCTIIIVAHRLSTIRQASKIVVINEGKVLEMGTHTELMDMKGAYHN  613
CpABC10            575 SVSGECTTIIVAHRLSTIRNANRIVVISAGKVQEIGSHAELMAKEGGYYN  624
TcABCB-3B          583 KASKGCTTVIVAHRLSTIRNANKIVVISKGKVVEQGTHNELMELKSEYYN  632
Dpon_c80822        573 KASQNRTTIIVAHRLTTIRGANKIVVISEGKVVEEGTHHELMKLQREYYT  622
Dpon_c4796A        578 RASENRTTIIVAHRLSTIRGANKIVVISDGKVVEQGTHEDLMELKKEYYT  627
Dpon_c306          566 RASEHRTTIIVAHRLSTIRGANKIVVISDGKVVEQGTHEDLMELKNEYYA  615
Dvv-isotig11620    576 AASVECTTIIVAHRLSTIRGANKIIVLSQGVVVEEGTHEELMELKQEYYR  625
Dvv-isotig07787    576 LASAECTTIIVAHRLSTIRGANKIIVLLEGTVVEQGTHDELMSLKGEYFK  625
CpABC12            578 SASVDCTTVIVAHRLSTIQGANKIMVFSKGAVVEQGTHDELMALKKEYYN  627
Ct-ABCB1           578 SASVDCTTVIVAHRLSTIQGANKIMVFSKGAVVEQGTHDELMALKNEYYN  627
                             * ..*****.**. * .*.    * * * *.*  **     *

TcABCB-3A          639 LVQSQGLVETEETT--TEEKQKQNGVVDTKPNQTEVTEIISTENLNDAQA  686
Dpon_c4796B        614 MITSQGYTDLKSENDKDNDKALQKSKSFSNHSRESAEDDQQVTEEEYFPQ  663
CpABC6             614 LVNSQGLMDTKPFS---QIQKIMRQVSSPMHQFVTDREDENVDEKSNDTQ  660
Dvv-isotig11596    614 LVISQGLTETLEEK---GNRRRSRKFSEANKS-INEKEDEELEDTQINQP  659
CpABC10            625 LVKSQGFTETQETT---GDRRLSRSFSLSNKSCIVEDEEEKIEDETAEES  671
TcABCB-3B          633 LVMTQVSAVEKFDGDQEGESRKLVELER-QVSLLDDEKHDDAEEEVQEA-  680
Dpon_c80822        623 LVTTQVQSGSQFESSTNEESKTEVAAAEDDEDEALSDKVEDEEAETDNY-  671
Dpon_c4796A        628 LVTTQVQGSEKFENIGETKEKKILDYAEDDDEDFAPKKEIDEEAEVDNY-  676
Dpon_c306          616 LVTTQVHSSEQFENIGEASEKKIFDSVEDDDEDFAPKRETDEESEVDNY-  664
Dvv-isotig11620    626 LVTAQVKSSEQFEVAEKKKVVRAISLAESSTGSD-HNIEATKEDNEDDFN  674
Dvv-isotig07787    626 LVTAQVASSNQIELSNDETETKGNNEAYEEYNDDDDDISDDEKNEIDDFK  675
CpABC12            628 LVTTQVKSKETVTQYTKSDKTQEHEDVIDEVVP--VEAAFAAEDDEDDFV  675
Ct-ABCB1           628 LVTTQVKSKETVTQYSKSDKTQEYDDDIDEVVP--VEASFAAEDDEDDFV  675
                       .. .*

TcABCB-3A          687 ENKGSPILQILKMNKPEWFHIFTGCVTAVINGSAFPIYGLVFGDIIGVLA  736
Dpon_c4796B        664 EGSSRTILKILRMNSSEWLSMIIGTLASFMNGASLPLYGLIFGDILGALS  713
CpABC6             661 VDKI--FIKVMKMCKPEWYLIAIGCVSSVIKGVAYPINGWIFGSIIGILF  708
Dvv-isotig11596    660 VAKN-ILWKVLKLNASEWFYILIGCLSSLITGASLPIYGLVFGGIMGIFA  708
CpABC10            672 VESKGVVLKVIKMNKPEWFYNFIGCLCSGLTGASLPVYGLVFGGIVGVLA  721
TcABCB-3B          681 -ERSVSLMSILRMNKPEWVSISIGCIASIVMGCSMPAFAVIFGDIMGVLA  729
Dpon_c80822        672 -VKKASIWSISKLNAPEWYLIVLGCVGAAAMGTSMPFFAVLFGNILTVLQ  720
Dpon_c4796A        677 -VKTASLWSIVKLNSPEWLSLVLGCIGAGAMGTAFPIFAILFGNILQVLQ  725
Dpon_c306          665 -VKTASLWSIVKLNSPEWLSLFLGCFGAAVMGTVLPIFAVLLGSILQVLQ  713
Dvv-isotig11620    675 ENKDVSVFEILKMNAPEWPYILFAGLGSIVVGCGMPVFAVLFGSILGTLA  724
Dvv-isotig07787    676 NGKKITLFSIMKLNAPEWPYILIAGVGSIVVGWGWPIFAVFFGSVLGTLA  725
CpABC12            676 SDRNMRLIDVIKMNAPEWPQIVVASIGSTVIGCAMPIFSVLFGSIIGTLA  725
Ct-ABCB1           676 SDRNMRLIDVIKMNAPEWPQIVVASIGSTVIGCAMPIFSVLFGSIIGTLA  725
                                . ..   **         .   *   *      * ..

TcABCB-3A          737 DPRDS--YVREQSNIFSLYFVIIGIVTAVATFLQIYYFAVAGEKLTKRLR  784
Dpon_c4796B        714 IIDNT--VLRREANFYCLYFLYLGIASGIAMFFQIYGFGYAGEKLTYRLR  761
CpABC6             709 LEEDD--ELLSENNTLCLYFICLAFIIGGSTFFQLLTFGIAGEKLTLRVR  756
Dvv-isotig11596    709 NDNDG--EVRSESNMYCLYFLILGVVTGVAMFWQTTSFSVAGEHLTLKIR  756
CpABC10            722 LTDDE--SVRRESNLYCLYFLILGIVTGIAMFFQMFSFGIAGEKLTRRIR  769
TcABCB-3B          730 EKNED--EVISETNRFCIYFVIAGVVSGIATFLQIFMFSVAGEKLTMRLR  777
Dpon_c80822        721 STDEE--YVRTQTNKYCLYFVYAGLMSMVATFMQMYMFGRAGQKLTYRIR  768
Dpon_c4796A        726 DIDSNPDYVRQETNKYCLYFVLAGVLSMCATFLQMYMFGRAGQKLTLRIR  775
Dpon_c306          714 DSDKN--YVRQETNKYILYFVLAGVLAMFATFLQLYMFGRAGENLTLRMR  761
Dvv-isotig11620    725 NGDPD--FVRSETNKYCLYFVLGGLITMVSVFTQMYLLGIAGEKMTERVR  772
Dvv-isotig07787    726 KYDTE--YIETETTRYCIFFVIAGVICMISVFLQNYLLGIAGEKMTERIR  773
CpABC12            726 DSKTE--YVRSETNKYVVYFVIAGAVAMVSVFLQMYMFGIAGEKMTERIR  773
Ct-ABCB1           726 NSDTE--YVRTETNKYVVYFVIAGAVAMVSVFLQMYMFGIAGEKMTERIR  773
                               .  . .   ..*.        . * *      **. .* ..*
```

```
TcABCB-3A          785 AKMFRAMLNQEMAWFDRKENGVGALCAKLSGEAASVQGAGGIRIGTVLNS 834
Dpon_c4796B        762 NKMFGCMLRQEMGWFDRKENGVGALCAQLSGDAASVQGAGGSRIGLILNS 811
CpABC6             757 LNTFRAILRQEMGWFDRKENGVGAICARLASDANNIQGLAGLHIGTVLNT 806
Dvv-isotig11596    757 SKTFEAMLRQEIGWYDQKSNGVGALCARLAGDAVAVQGAAGPQIGTTINF 806
CpABC10            770 HKTFEAMMNQEMGWFDKKENGVGALCAQLAGDAAAIQGVAGVQIGTVLNF 819
TcABCB-3B          778 SMTFIAMLKQEMGWYDRKDNGVGALCARLSGEAAHVQGATGQRVGTILQS 827
Dpon_c80822        769 SRMFEALLKQEMAYYDRKSNGVGSLCAKLSDEAASVQGATGQRIGSIMSS 818
Dpon_c4796A        776 SRMFDALLKQEMGYFDRKENGVGSLCAKLSNEAAQVQGATGQRIGTIVNS 825
Dpon_c306          762 SRMFDALLKQEMGYFDRNENGVGSLCAKLSNEASQVQGATGQRIGAIVNS 811
Dvv-isotig11620    773 SRLFKAMIYQEIGFFDKKTNGVGALCAKLSSDASNIQGATGIRVGTILQS 822
Dvv-isotig07787    774 TQMFTAIISQEMGFFDKKSNGVGALCAKLAGDSSSIQGATGQRVGAILQS 823
CpABC12            774 GKMFSAMLNQEIGFFDKKSNGVGALCAKLSSDAASVQGATGQRIGVVLQS 823
Ct-ABCB1           774 GKMFSAMLNQEIGFFDKKTNGVGALCAKLSSDAASVQGATGQRVGVVLQS 823
                          * ... **.  .*.  ****..**.*. ..  .**  * ..*  .

TcABCB-3A          835 LATFIISNIIALYFEWRLALVLISFSPIILLSVFFEQKFTQGDSQVNQKY 884
Dpon_c4796B        812 VSTFILAACIGFYLEWRLTLVAGVFFPLMFFSISYERKSVQQETQAAQKL 861
CpABC6             807 LTTFTVTLVFCFYYEWKLTLVLIAIFPLIFLSVYLEQKFLQDDIIKNQLM 856
Dvv-isotig11596    807 ISTFILTCTFSFYFEWRTSFVLFSLCPVIFFSVYFEQKVLQEDATKNQKM 856
CpABC10            820 LCTFILTCAFSFYYEWKLTLVLLSFCPLIFFSVYFEQKSMQNDAIKNQKM 869
TcABCB-3B          828 IATIGLSVGLSMYYQWKLGLVALAFTPFILLAVFFQHRLMNVENEAHHKS 877
Dpon_c80822        819 LSTLAFALFLALFYQWRLGLVALSFFPLILISTFFQRRQMSQESDEYRAS 868
Dpon_c4796A        826 LATLSLSVFLAVYYEWRLGLVAMVFVPLIIVATFLQRRQMSQESDDYKES 875
Dpon_c306          812 LATLILSVFFAVYYEWRLGLVAMVFVPLIIAATFIQKKQMSQESDDYKES 861
Dvv-isotig11620    823 IATFCLAIGLSMYYEWKLGLVTAAFTPVILIAMFFERRNTRGGNDSRDSA 872
Dvv-isotig07787    824 LSTFGIAIALSMYYQWKLGLLMASFTPLMLVAIFIEKRNTTGLSEAREKS 873
CpABC12            824 MATFCLAVGLAMYYEYRLGLVTVAFMPFLLIAFFFERRNSSGQNDTRDKA 873
Ct-ABCB1           824 MATFCLAVGLAMYYEYRLGLVTVAFMPFLLIAFFFERRNSSGQNDTRDQS 873
                       ..*   .     . . .   .     * .  .   ...

TcABCB-3A          885 LENSAKIAVEAIGNIRTIASLGCEEVFHGYYVKELTPYVANV-KKQMHFR 933
Dpon_c4796B        862 LEKSAKIAIEAIDNIKTVKALGCERVFCDTYEKELDLCRQAG-FKRSHIK 910
CpABC6             857 LEKSSKLAIEAIGNIRTVVSLGCEQVFLDLFVKELLPYRAMA-NRKSHMR 905
Dvv-isotig11596    857 LEASAKLAVEAIGNIRTVVSLGCEKVFMEQYIKELLPYQKMA-RKKSHYR 905
CpABC10            870 VERSSKLAVEAIGNIRTVVSLGCERVFHDMYVKELSPYQALA-RRKSHFR 918
TcABCB-3B          878 LQKSNKLAVEAVGNVRTVVSLGLEETFHKLYISYLMEHHKRT-LRNTHFR 926
Dpon_c80822        869 LQKSTKIAVEAVGSIRTVVSLGCEDIFYNLYISELMPHIKTC-LRNSHGR 917
Dpon_c4796A        876 LQKSTKIAVEAVGSIRTVVSLGCEETFLNLYINELTPHIKKC-LRNTHAR 924
Dpon_c306          862 LEKSTKIAVEAVGSIRTVVSLGCEKTFFKLYISELMPHIKKC-LRNTHAR 910
Dvv-isotig11620    873 LQKSTRTAVEAVGNIRTVASLGLEEKFQQLYESELMPHYKSS-LKTVHWR 921
Dvv-isotig07787    874 LQKSTKIAVEAVGNIRTVAGLVAEDKFQKSYITELKPHYKAA-LAAVHWR 922
CpABC12            874 LQKSTKIAVEGVGNIRTVASLGLEEKFHHLYISELMPHYKNSNRASLHWR 923
Ct-ABCB1           874 LQKSTKIAVEGVGNIRTVASLGLEEKFHHLYISELLPHYKNSSSASLHWR 923
                       .. * . *.* .  ..*.  *  *  *   .   *            * .

TcABCB-3A          934 SAVLGVARSVMLFAYAVGMGYGAKLMVDSDVDYGTVFIVSETVIVGSWSI 983
Dpon_c4796B        911 AGLIGMARCIQFLAYAGGMTYGAQLLEQNEVDSATLFKVLEVIVTSSWSI 960
CpABC6             906 GLILGMARALMIASYLAGIMYGGKIIIDGEAEYGTVFKVCEIMTMGSWAI 955
Dvv-isotig11596    906 GIIVGLARSLMLFAYVAGIRYGINLIISGDCPYGTIFIVCEVMIVGTWSV 955
CpABC10            919 GTVLGMARSLIVFAYAAGMGYGIKLIIDGAVEYGIVIKVCEVMIVGSWSI 968
TcABCB-3B          927 AVVLGLARSIMFFAYSACMYYGGHLIRDEGLLYQDVFKVSQSLIMGTVSI 976
Dpon_c80822        918 ALVLAFSRAILLFAISACLYYGGHLIRDGQVEFGDVFKVTQGLVMGTTSI 967
Dpon_c4796A        925 AFILGFSRAIMIFAFSACLVYGGYLIKNDNVQYGDVFKVAQALIMGTVSI 974
Dpon_c306          911 AFILGFSRAIMIFAFSACLVYGGYLIKHENVQYGVVFKVAQALIMGTVSI 960
Dvv-isotig11620    922 AIVFGLSRSLLFFAYATAMYYGGFLIR-DGLPYDRVFKVSQAQIMGTVSI 970
Dvv-isotig07787    923 GLVFGLSRSLGYFAYAAAMYYGGFLIR-DGLYYDKVFKVAQAQLMGTISI 971
CpABC12            924 GIVFGLSRGLSFFAYSAAMYYGGYLIKNDNLSYEKVFKVSQALIMGTTSI 973
Ct-ABCB1           924 GIVFGLSRGLSFFAYSAAMYYGGYLIKNENLSYEKVFKVSQALIMGTTSI 973
                         .   .* .   .    . **  ..         .  * .     . ..
```

```
TcABCB-3A        984 GNAFSFSPNFQKGLSAADRIFSLLKRVPEVKNSLEPVYLNDVRGNIEYSN 1033
Dpon_c4796B      961 GNALSFSSNMQKGITAAAKIFRLLNREPAIKNSPNGIVRYLEKADVEYSK 1010
CpABC6           956 GNALALPPNFQQALNAAARTISLLERKPSVKTTGNP----WRDGNVEYND 1001
Dvv-isotig11596  956 GNALSLSPNFQKGLVAASRIITLLERQPVVQNMPDALNFLWEDENVEYSE 1005
CpABC10          969 GNAMSMTPNFQKGLIAARRIISLLDRTPLVKNIANPSKKLWENENIEFSQ 1018
TcABCB-3B        977 ANALAFTPNLQKGLVAAARIIRLLRRQPLIRDEPGAKDKEWENGAIQYDT 1026
Dpon_c80822      968 ANSLAFTPNLEKGLIAAKSVMNTINRITQIRNLPDAKNKTQAEGDLGYSD 1017
Dpon_c4796A      975 ANSLAFTPNLEKGLVAARTVMNMINRIPKVSNSQNALIKKTADGNVDYSQ 1024
Dpon_c306        961 ANSLAFTPNFEKGLVAARTVMNMINRIPKVSNSQNALIKNTADGNVNYSQ 1010
Dvv-isotig11620  971 ANSLAFSPNFAKGVAAAKKVKSFLSRIPLIRDLPSSRQMVKASGNFSFSE 1020
Dvv-isotig07787  972 ANSLAFTPNLNRGISAAKRIKSFLSRIPLIRDGPSSKPMNQVDGNIGYSN 1021
CpABC12          974 ANALAFTPNFTKGLSAAKSVQKFLERVPKIRDDMNSKDVNEVEGDISFSK 1023
Ct-ABCB1         974 ANALAFTPNFTKGLNAAKSVQKFLERMPKIRDDMNSKDVNEVEGDISFAK 1023
                      *. .   *  . . **      . *   .                 .

TcABCB-3A       1034 IYFSYPTRSSVSVLNGLNLNVLQGKTVALVGASGCGKSTIIQLLERFYDP 1083
Dpon_c4796B     1011 IYFSYPTRPAIPILNGLDLSILNGKTVALVGGSGCGKSTLIQLLIRFYDP 1060
CpABC6          1002 IFFSYPTRPSISVLNGLDLRVMQGKTVALVGSSGCGKSTVVQLLERFYDP 1051
Dvv-isotig11596 1006 VYFSYPTRPSIPILKALNLLIPKGKTVALVGSSGCGKSTIIQLLERFYDP 1055
CpABC10         1019 IYFSYPTRPSISILNSLNLSILKGKTVALVGSSGCGKSTIIQLLERFYDP 1068
TcABCB-3B       1027 IYFSYPTRPNIMVLKGLNLSVLQGKTVALVGPSGCGKSTIIQLIERFYDP 1076
Dpon_c80822     1018 VHFSYPTRVKIPVLRGLDLSVLRGKTVALVGPSGCGKSTIVQLLERFYDP 1067
Dpon_c4796A     1025 IRFAYPTRDSIQVLKGLDLSVLQGKTVALVGPSGCGKSTIIQLLERFYDP 1074
Dpon_c306       1011 IHFSYPTRDSIEVLKGLDLSVLQGKTVALVGPSGCGKSTIIHLLERFYDP 1060
Dvv-isotig11620 1021 IEFTYPTRPNVLILKGLNLDILNGKTVALVGESGCGKSTIIQLIERFYDP 1070
Dvv-isotig07787 1022 VEFFYPTRKNIQVLRGLTINIPKGSTVALVGESGCGKSTIIQLIERFYDP 1071
CpABC12         1024 IKFAYPTRPGTMVLRDLDLKIFKGKTVALVGQSGCGKSTLIQLIERFYDP 1073
Ct-ABCB1        1024 IKFAYPTRPGTTVLRDLDLRIFKGKTVALVGQSGCGKSTLIQLIERFYDP 1073
                     . * ****    .*  * . .  * ****** *******...*. *****

TcABCB-3A       1084 VSGEVSLDGESVKTVDIQNLRSHLGIVSQEPNLFDRTIAENIAYGAND-R 1132
Dpon_c4796B     1061 GYGEVAIGGDDVRALSLKHLRSHLGIVSQEPNLFDLTIAENISYGIHD-R 1109
CpABC6          1052 AGGAITVDGVDTRSMRLGQLRSQLGIVSQEPNLFDRTVAANISYGAVEEK 1101
Dvv-isotig11596 1056 SYGKVEISDKNIRYVDLKSLRSQLGIVSQEPNLFDKTIAENIAYGANY-K 1104
CpABC10         1069 DYGQITVDGEDTTKTDMATMRSQLGIVSQEPNLFDRTIAENISYGANQ-K 1117
TcABCB-3B       1077 LEGTLTVDNEDIRNIRLGSHRSHLGIVSQEPNLFDRTIGDNIAYGDNS-R 1125
Dpon_c80822     1068 LSGSVTLDRGDLKSLTLSSLRSHLGIVSQEPNLFSRTIAENIAYGDNA-R 1116
Dpon_c4796A     1075 ASGTVSLDKDDIKSITLDSLRSHLGIVSQEPNLFSRSIAENIAYGDNS-R 1123
Dpon_c306       1061 ASGTVSLDEDDIKSITLASLRSHLGIVSQEPNLFSRSIAENIAYGDNS-R 1109
Dvv-isotig11620 1071 RSGEVKMDGVDLKDISLDSLRSHMGIVSQEPNLFNKSIAENIAYGDNS-R 1119
Dvv-isotig07787 1072 SSGDINLDEQNIKHITLSSLRSHLGIVPQEPNLFNKSIAENIAYGDNS-R 1120
CpABC12         1074 SEGEVMLDEINVKRMRLSSLRSHLGIVSQEPNLFNKTIRENISYGDNG-R 1122
Ct-ABCB1        1074 TGGEVMLDDIDVKRMKLRSLRSHLGIVSQEPNLFNKTIRENISYGDNG-R 1122
                       * . .         .   **..*** ******  ..  **.**    .

TcABCB-3A       1133 TVGMNEIVEAAKSANIHTFISSLPGGYETSLGSKGAQLSGGQKQRVAIAR 1182
Dpon_c4796B     1110 KVDLKEIMEAAKSANVHSFVTSLPLGYETRLGSKGKQLSGGQKQRIAIAR 1159
CpABC6          1102 DARIDRIMEAAKSANIHTFISSLPMGYDTRVGTNGTQLSGGQKQRIAIAR 1151
Dvv-isotig11596 1105 QVEMDAIIDAAKSANIHEFILNLPKGYETKVGSKGTQLSGGQKQRIAIAR 1154
CpABC10         1118 EVDMEKVMESAKSANIHNFITALPMGYETRIGSNGTQLSGGQKQRIAIAR 1167
TcABCB-3B       1126 EVTQEEIIEAAKNANIHNFIASLPLGYETRLGEKGTQLSGGQKQRVAIAR 1175
Dpon_c80822     1117 TVPMAEIIEAATKANIHTFITGLPKGYDTKLGDKGTQLSGGQKQRIAIAR 1166
Dpon_c4796A     1124 QATEVEIIEAARKANIHNFIAHLPKGYKTKLGEKGTQLSGGQKQRIAIAR 1173
Dpon_c306       1110 HSTDSEIIEAARKANIHNFITQLPEGYNTKLGEKGTQLSGGQKQRIAIAR 1159
Dvv-isotig11620 1120 EVSMDEIIKAAKNANIHNFITGLPKGYETKLGEKAVQLSGGQKQRIAIAR 1169
Dvv-isotig07787 1121 NVPIDEIIQAAKNANIHNFIVGLPNGYDTELGEKATQLSGGQKQRIAIAR 1170
CpABC12         1123 VVQMDEVIQAAVNANIHNFISGLPKGYETSLGEKAVQLSGGQKQRIAIAR 1172
Ct-ABCB1        1123 VVQMDEVIQAAVNANIHTFISGLPKGYETTLGEKAVQLSGGQKQRIAIAR 1172
                           .. .*  **.* *.  ** ** * .*    *********.****
```

```
TcABCB-3A       1183 ALIRNPKILLLDEATSALDNESEKVVQEALDNAKKNRTCITIAHRLTTIQ 1232
Dpon_c4796B     1160 ALLRDPKILLLDEATSALDNESEKIVQEALDNARKGRTCITIAHRLTTIQ 1209
CpABC6          1152 ALMRNPKILLLDEATSALDNESKKIVQQALEVARKGRTCITIAHRLTTIQ 1201
Dvv-isotig11596 1155 ALLRNPKILLLDEATSALDNESEKIVQEALDNARKSRTCITIAHRLTTIQ 1204
CpABC10         1168 ALMRNPKILLLDEATSALDNESEKIVQEALDNARQGRTCITIAHRLTTIQ 1217
TcABCB-3B       1176 ALVRNPKLLLLDEATSALDSESEKVVQEALDNAKKGRTCITIAHRLTTIQ 1225
Dpon_c80822     1167 ALVRNPKVLLLDEATSALDAESEKVVQEALDNAKQGRTCLTIAHRLTTIQ 1216
Dpon_c4796A     1174 ALIRQPKVLLLDEATSALDAESEKVVQEALDNAKQGRTCLTIAHRLTTIQ 1223
Dpon_c306       1160 ALIRHPKVLLLDEATSALDAESEKVVQDALDNAKQGRTCLTIAHRLTTIQ 1209
Dvv-isotig11620 1170 ALVRNPKVLLLDEATSALDTESEKVVQEALDQAKKGRTCVTIAHRLTTIQ 1219
Dvv-isotig07787 1171 ALVQNPKILLLDEATSALDTESEKVVQAALDEAKKGRTCITIAHRLTTIQ 1220
CpABC12         1173 ALVRNPKVLLLDEATSALDTESEKIVQEALDQAKMGRTCITIAHRLSTIQ 1222
Ct-ABCB1        1173 ALVRNPKVLLLDEATSALDTESEKVVQEALDQAKLGRTCITIAHRLSTIQ 1222
                     **.. **.*********** ** *.** **. *.  ***.******.***

TcABCB-3A       1233 DADLICVLNEGVVAEMGKHNELLDKKGLYYDFYKLQTGQK   1272
Dpon_c4796B     1210 DADVICVVKEGQIAEMGTHGELLKLKGHYYDYYKMQSGQN   1249
CpABC6          1202 DADLICVLEKGMVAEMGDHQTLLQRRGLYYEFYKLQTCQ    1240
Dvv-isotig11596 1205 DADVICVLKEGNVVEMGTHKELLEKQGLYYKFYKLQSVESIS 1246
CpABC10         1218 DADVICVLKEGFIAEMGTHTELLIKRGMYYKFYKLQAGQT   1257
TcABCB-3B       1226 DADVICVIDKGVVAEIGTHSELLSQKGLYYKLHSLQNK     1263
Dpon_c80822     1217 DADVICVVNEGVIAEQGTHSELIEKKGLYYKLHALQH      1253
Dpon_c4796A     1224 DADLICVVNDGVIVEQGNHTDLIERKGLYYRLHAHQH      1260
Dpon_c306       1210 DADLICVVNNGVIVEQGAHTDLIERNGLYYRLYARQHYFL   1249
Dvv-isotig11620 1220 DADLICVVANGVIAESGSHQELLQKEGLYYKLYTQKT      1256
Dvv-isotig07787 1221 DADIIYVIDKGIVVESGTHRELLNKNGFYYKLYTQKH      1257
CpABC12         1223 DADMICVIDRGIVAEAGTHAELLEKKGLYYKLQRQAT      1259
Ct-ABCB1        1223 DADMICVIDRGIVAEAGTHAELLEKKGLYYKLQRQTT      125
```

Figure S5. CLUSTAL Alignment of ABCB protein sequences from Coleoptera. CRW424: Chromosome 8 marker linked to Cry3Bb resistance in *Diabrotica virgifera virgifera* (Ref. [17]). Other abbreviations as in Figure S4. * The asterisks denote identical amino acids and the dots denote conserved amino acids.
